# MAP3K19 Is a Novel Regulator of TGF-β Signaling That Impacts Bleomycin-Induced Lung Injury and Pulmonary Fibrosis

**DOI:** 10.1371/journal.pone.0154874

**Published:** 2016-05-04

**Authors:** Stefen A. Boehme, Karin Franz-Bacon, Danielle N. DiTirro, Tai Wei Ly, Kevin B. Bacon

**Affiliations:** 1 AxikinPharmaceuticals, Inc., San Diego, California, United States of America; 2 DNA Consulting, Inc., San Diego, California, United States of America; Children's Hospital Los Angeles, UNITED STATES

## Abstract

Idiopathic pulmonary fibrosis (IPF) is a progressive, debilitating disease for which two medications, pirfenidone and nintedanib, have only recently been approved for treatment. The cytokine TGF-β has been shown to be a central mediator in the disease process. We investigated the role of a novel kinase, MAP3K19, upregulated in IPF tissue, in TGF-β-induced signal transduction and in bleomycin-induced pulmonary fibrosis. MAP3K19 has a very limited tissue expression, restricted primarily to the lungs and trachea. In pulmonary tissue, expression was predominantly localized to alveolar and interstitial macrophages, bronchial epithelial cells and type II pneumocytes of the epithelium. MAP3K19 was also found to be overexpressed in bronchoalveolar lavage macrophages from IPF patients compared to normal patients. Treatment of A549 or THP-1 cells with either MAP3K19 siRNA or a highly potent and specific inhibitor reduced phospho-Smad2 & 3 nuclear translocation following TGF-β stimulation. TGF-β-induced gene transcription was also strongly inhibited by both the MAP3K19 inhibitor and nintedanib, whereas pirfenidone had a much less pronounced effect. In combination, the MAP3K19 inhibitor appeared to act synergistically with either pirfenidone or nintedanib, at the level of target gene transcription or protein production. Finally, in an animal model of IPF, inhibition of MAP3K19 strongly attenuated bleomycin-induced pulmonary fibrosis when administered either prophylactically ortherapeutically. In summary, these results strongly suggest that inhibition of MAP3K19 may have a beneficial therapeutic effect in the treatment of IPF and represents a novel strategy to target this disease.

## Introduction

Idiopathic pulmonary fibrosis (IPF) is a chronic, disabling lung disease with a median survival time of only 2–3 years after diagnosis [1]. IPF progression is characterized by normal lung parenchyma becoming progressively replaced with fibrotic tissue, which leads to dyspnea, cough, impaired lung function and ultimately death [1–3]. The pathogenesis of IPF is poorly understood, however the initial pathology driving the disease process is postulated to be an aberrant repair mechanism in response to repetitive alveolar epithelial cell (AEC) injury [2]. The causes of AEC injury remain to be identified, but cigarette smoke, inhaled particulates or other environmental exposures, viral infection and gastroesophageal reflux have all been hypothesized to be triggers [2, 4, 5]. Alveolar epithelial cell death initiates a wound healing response including fibroblast recruitment to the pulmonary tissue. These cells then proliferate and differentiate into myofibroblasts, which are considered the hallmark cells of IPF [6]. The myofibroblasts form foci, and the fibrosis they produce may occur in both the pulmonary interstitium and the airspaces, and lead to thickened fibrotic bands in the lung [7].

Multiple hallmarks of aging have been identified in IPF tissue, such as genetic instability and telomere attrition [8]. It has been postulated these aging-associated hallmarks may contribute to both chronic obstructive pulmonary disease (COPD) and IPF, causing the respective disease in patients exhibiting a predisposition [7, 9, 10]. Further, COPD and emphysema are often associated comorbidities in patients with IPF [11, 12].

Until very recently, therapeutic efforts to treat patients with IPF have been disappointing. However in 2014, two drugs, pirfenidone and nintedanib, received approval for treatment of IPF and have quickly become the standard of care [13–15]. Both compounds have anti-fibrotic properties and have been shown to reduce the functional decline and disease progression in IPF patients with mild to moderate functional impairment [16, 17]. Both pirfenidone and nintedanib cause gastrointestinal side effects and elevations in liver-associated enzymes. In addition, pirfenidone is associated with increased photo-sensitivity [13–17]. Thus, these drugs have associated risks and side effects, and cannot reverse the progression of IPF.

A multitude of profibrotic mediators have been implicated in the pathogenesis of IPF and the pleiotropic cytokine TGF-β has been shown to be a central mediator [18, 19]. TGF-β has the ability to attract fibroblasts and stimulate their proliferation, as well as to induce the epithelial to mesenchymal transition (EMT) of alveolar epithelial cells [20]. TGF-β-induced activation of the receptor complex on target cells leads to activation of the receptor-regulated effector proteins (R-Smads) Smad2 and Smad3 through direct C-terminal phosphorylation of an SSXS motif [21, 22]. Phosphorylated Smad2 and Smad3 then form trimers with Smad4, and translocate into the nucleus, where they associate and cooperate with DNA binding transcription factors to activate or repress target gene transcription [23, 24]. For example, genes such as collagen, fibronectin and N-cadherin are upregulated while claudins, occludin and E-cadherin are down-regulated, thereby contributing to EMT and the fibrotic phenotype [25].

MAP kinases control a wide range of cellular activities and are particularly important in controlling gene expression and programmed cell death in a wide variety of cell types throughout the body [26]. They are able to regulate these cellular functions by phosphorylating substrate proteins, such as other protein kinases, transcription factors and cytoskeletal proteins. MAP kinase signaling pathways comprise a three component protein kinase cascade of serine-threonine protein kinase (MAP 3-kinase), which phosphorylates and activates a dual-specificity protein kinase (MAP 2-kinase) that, in turn, phosphorylates and activates a MAP kinase. The activity of MAP 3-kinases are primarily regulated by phosphorylation, often by cell surface receptors [27]. In addition to kinase regulation, phosphorylation may also control interactions of MAP 3-kinases with regulatory proteins and downstream MAP kinases [27, 28].

We report here on the expression and activity of MAP3K19, also referred to as YSK-4, RC kinase or RCK (patent number WO 2005/001083 A1; protein accession number Q56UN5). We first became interested in MAP3K19 because of our initial observation that it was over-expressed in the lungs of COPD patients. As IPF is a co-morbidity of COPD and shares some pathological similarities, such as an aging phenotype in affected cells, we explored the role of MAP3K19 in IPF. We show that MAP3K19 has a limited tissue expression pattern and plays a role in regulating TGF-β-induced Smad signaling and gene expression. Finally, antagonism of MAP3K19 by a small molecule inhibitor, or siRNA, can inhibit TGF-β signaling and target gene transcription as well as potently diminish bleomycin-induced lung fibrosis in a murine model of IPF.

## Materials and Methods

### Materials

All reagents were purchased from Sigma-Aldrich Chemical Co. (St. Louis, MO) unless otherwise stated. Pirfenidone and nintedanib were purchased from Selleckchem (Houston, TX). TGF-β1 was purchased from R&D Systems (Minneapolis, MN) and reconstituted according to their instructions. A panel of human organ RNAs was purchased from Clontech Laboratories, Inc. (Mountain View, CA). Human lung tissue was obtained from Analytical Biological Services, Inc. (Wilmington, DE). Human tissue microarrays were purchased from Protein Biotechnologies (Ramona, CA). Compound A is a proprietary, highly potent and selective MAP3K19 small molecular weight inhibitor that was developed as part of the Axikin Pharmaceuticals, Inc. portfolio of compounds (US Patent number 60/096308).Inhibition assays examining the activity of Compound A on the kinase activity of MAP3K19 showed an IC_50_< 20 nM. Additionally, in vitro specificity testing showed that 1 μM of Compound A did not inhibit greater than 50% of the kinase activity of 300 protein kinases.

### Gene Expression Analysis

RNA was isolated from cells using the RNeasy kit (Qiagen, Valencia, CA), and total RNA was reverse transcribed using SuperScript III (Life Technologies, Carlsbad, CA). Fifty nanograms of cDNA was quantified using the 7900HT Fast Real-Time PCR system (Applied Biosystems, Inc., Foster City, CA) using Power SYBR Green PCR Master Mix (Applied Biosystems, Inc.) and performed by the CFAR Genomics and Sequencing Core of UCSD/VMRF (San Diego, CA). Each sample was run as a technical duplicate. The oligonucleotide primers (125 nM final concentration) were designed and validated by Integrated DNA Technologies (San Diego, CA), with the exception of MAP3K19 primers, which are as follows: sense primer: 5’ aatggcacccacagtgacatgctt 3’; anti-sense primer: 5’ ccctcggtgtgctccgatgtaaaa 3’. The amplification efficiencies were determined for each target gene and normalized to human GAPDH. The delta Ct was calculated using the comparative analysis method.

### Histological Analysis

Two antibodies directed against different MAP3K19 epitopes were produced by Pro-Sci Inc. (Poway, CA). 1B9C2 is a mouse IgG1 monoclonal antibody and RabK19 is a rabbit IgG polyclonal antibody. Histological analysis was performed by Reveal Biosciences, Inc. (San Diego, CA) and all antibodies used in this study were validated and titrated by Reveal Biosciences. Briefly, immunohistochemical analysis of normal adult lung sections or IPF lung sections (kindly provided by Dr. Cory Hogaboam, whose present address is Cedars-Sinai Medical Center, Los Angeles, CA) was performed on a Leica Bond automated immunostainer (Leica Biosystems, Inc., Buffalo Grove, IL). The primary antibody was detected by Bond Polymer Refine Detection System (Novacastra Reagents, Leica Biosystems Inc.) with 3’3’-diaminobenzidine (DAB, brown) as the substrate and a hematoxylin nuclear counterstain (blue). Whole slide imaging was performed in bright field using a Pannoramic Scan from 3D Histech (Perkin Elmer, Waltham, MA).

For staining of normal human lung with anti-MAP3K19 (RabK19), anti-CD68 (Origene Technologies, Rockville, MD) and anti-surfactant protein C (BiOrbyt, Cambridge, UK), human lung sections obtained from BIO Options (Brea, CA) were dewaxed and antigen retrieval was performed using a 1X DIVA Decloaker (BioCare Medical (Concord, CA) for twenty minutes. Non-specific protein binding was blocked with 3% Normal Donkey Serum in PBS + 0.1% Triton X-100 (Sigma-Aldrich Chemical Co.). Slides were stained with each primary antibody (all at 1:100) and with secondary antibodies at 1:200 (AlexaFluor 546 donkey anti-rabbit and AlexaFluor 647 donkey anti-mouse, Life Technologies). Slides were mounted using Fluoro Gel II with DAPI (Electron Microscopy Services, Hatfield, PA). Slides were imaged as described above.

For immunofluorescence studies with HeLa cells, the cells were grown overnight on glass coverslips in 6-well dishes and treated as described. At the appropriate time, the cells were fixed in 4% paraformaldehyde, blocked in 3% normal donkey serum plus 0.1% Triton X-100 and incubated in primary antibodies overnight at 4°C. The primary antibodies were RabK19 rabbit anti-MAP3K19 and anti-phospho-Smad2/3 (BD Biosciences, Franklin Lakes, NJ). Primary antibodies were detected using AlexaFluor 546 donkey anti-rabbit and AlexaFluor 647 donkey anti-mouse secondary antibodies, and mounted in Fluor-Gel II with DAPI. The cells were then imaged as described.

The rapid IPF patient was diagnosed using a multidisciplinary approach involving clinicians, radiologists, and pathologists. The IPF patient was retrospectively grouped into a rapidly progressive group based on the following criteria during the first year of follow-up: mortality or acute exacerbation, percent forced vital lung capacity (FVC) decrease of ≥ 10%, and percent diffusing capacity of carbon monoxide (DL_CO_) decrease of ≥ 15%. A University of Michigan Institutional Review Board approved this study and written informed consent was obtained from each patient. The IRB approval number from the University of Michigan is HUM0095-304.

### Isolation of Human Bronchoalveolar Lavage Macrophages

Bronchoalveolar lavage (BAL) from human patients was approved and performed at the Universita degli studi di Roma Tor Vergata under the supervision of Dr. Cesare Saltini. The protocol for human subjects and material obtained was part of a collaborative project between ReDD (Research for Drug Discovery) Tor Vergata,the Cattedra Malattie Apparato Respiratorio & Centro Interdipartimentale per lo Studio delle Malattie Rare del Polmone e della Fibrosi Polmonare, Facoltà di Medicina e Chirurgia of the University of Rome, and Axikin Pharmaceuticals, Inc. Patients provided written consent for this study, and the consent procedure and investigational protocol was approved by the Policlinico Universitario (University Hospital) Tor Vergata Ethics Committee (SCS/REDD/08 n. 123/08).BAL macrophages were isolated from four healthy patients, 6 healthy smokers and 5 patients with mild-to-moderate idiopathic pulmonary fibrosis. None of the patients were on immunosuppressive or anti-fibrotic therapy at the time of the study. The cells from the BAL lysates were assayed for viability by trypan blue cell counts and cell purity by flow cytometric analysis or Diff Quick (Thermo Fisher Scientific, Waltham, MA) stained cytocentrifuged slide preparations. Samples that had greater than 5% ciliated epithelial cells were discarded. Alveolar macrophages were further purified by adherence to plastic tissue culture dishes (Corning, Thermo Fisher) for thirty minutes at 37°C. RNA was then isolated from the purified BAL macrophages (>95% purity) using the RNA/DNA/Protein Purification kit from Norgen Biotek (Thorold, Ontario, CAN) and used for RT-qPCR as described previously [29].

### Cell Culture

A549, HeLa and THP-1 cells were purchased from the ATCC (Manassas, VA) and cultured according to ATCC instructions. Briefly, F12-K (A549) and DMEM (HeLa and THP-1) (both medias from ATCC) base medias were supplemented with 10% FCS (Life Technologies), 2 mM glutamine (Corning Life Sciences, Tewksbury, MA), 10 mM HEPES (Corning Life Sciences), 100 μM non-essential amino acids (Corning Life Sciences), sodium pyruvate (Corning Life Sciences), penicillin and streptomycin (Corning Life Sciences). Cells were transfected with siRNA directed against MAP3K19 or non-sense siRNA. The sequence of the MAP3K19 siRNA was 5’ AGCATTGGTTGTACTGTGTTT 3’, and the non-sense siRNA sequence was 5’ TCCATAACGCGTATACTCGAC 3’(Qiagen Sciences) using the Lipofectamine RNAiMAX Reagent (Life Technologies). Cells were also transfected with a kinase dead mutant of MAP3K19 (K1089R mutation) generated by using the QuikChange II XL Site-Directed Mutagenesis Kit (Agilent Technologies, Santa Clara, CA) and the Effectene Transfection Reagent (Qiagen Sciences). A549 cells were also stably transfected with a Smad luciferase reporter plasmid that contains three copies of a Smad-binding element (SBE) that drives transcription of luciferase reporter gene (pGL4.48, Promega Corp., Madison, WI). Luciferase activity was measured using the One Glo Luciferase Assay System (Promega Corp.) according to the manufacturer’s instructions.

### Western Blotting& Protein Analysis

Cells were treated as described, and nuclear and cytoplasmic extracts were made using the NE-PER Nuclear and Cytoplasmic Extraction Reagents (Pierce, Rockford, IL) in the presence of protease and phosphatase inhibitors (Halt Protease and Phosphatase Inhibitor Cocktail, Pierce). Total protein concentration was determined in each sample using the BCA Protein Assay Kit (Pierce). Western blots were essentially done as previously described [30]. Twenty micrograms of protein was electrophoresed through 10% polyacrylamide gels (Life Technologies) and transferred to nitrocellulose using the iBlot dry transfer system and reagents (Life Technologies). Following a one hour blocking step in 10% BSA (fraction V, Sigma-Aldrich) and TBST buffer, the blots were hybridized overnight. All antibodies were purchased from Cell Signaling Technologies (Danvers, MA), except the MAP3K19 rabbit polyclonal antibody RabK19 (used at 1:250). The Cell Signaling Technologies antibodies were diluted (1:1000) and included anti-phospho-Smad2 (Ser 465/467, Cat. #3101), anti-phospho-Smad3 (Ser 423/425, Cat. #9520), anti-Smad4 (Cat. #9515) and anti-HDAC-1 (Cat. #2062).Western blots were developed using SuperSignal West Femto Maximum Sensitivity Substrate (Pierce) and visualized using a Kodak Gel Logic 440 Imaging System (Eastman Kodak, New Haven, CT). Cytoplasmic cell lysates were assayed for PAI-1 (Serpine-1) protein levels using a PAI-1 ELISA according to the manufacturer’s instructions (R&D Systems).

### Bleomycin-Induced Pulmonary Fibrosis Model

All animal experiments were approved by and carried out in strict accordance of the Institutional Ethics Committee of Animal Care of Preclin Biosystems AG (Epalinges, Switzerland). All efforts were made to minimize animal suffering, and at the termination of the study, mice were sacrificed by lethal intraperitoneal (i.p.) injection of sodium pentobarbital in accordance with the Ethics Committee guidelines. C57BL/6J female mice (Charles River Laboratories, Leiden, Netherlands) were 8 weeks of age at the start of the study and maintained in specific pathogen free environment for the duration of the study. On day 0 of the studies mice were administered 3 U/kg bleomycin (bleomycin sulfate, Merck Chemicals, Schaffhausen, Switzerland) via intra-tracheal (i.t.) instillation. For the prophylactic study, bleomycin-treated mice received oral administration of either Compound A (10 mg/kg, bid, 12 mice), Compound A vehicle alone (80% Solutol, 20% ethanol, Sigma-Aldrich; 12 mice), or dexamethasone (3 mg/kg every two days, delivered i.p.; Sigma-Aldrich; 12 mice), or were untreated (5 mice). All treatments for the prophylactic study started on Day 0 and extended to Day 13. For the therapeutic study, there were 6 groups of mice, and the treatment groups were (1) a saline treated control group (5 mice); (2) bleomycin treated control group with no further treatment (5 mice); (3) bleomycin treated mice treated with vehicle control(80% Solutol, 20% ethanol;12 mice); (4) bleomycin treated mice treated with Compound A (10 mg/kg, bid, 12 mice); (5) bleomycin treated mice treated with pirfenidone (10 mg/kg, bid, administered in 0.5% carboxymethylcellulose sodium, Sigma Aldrich; 12 mice) and (6) bleomycin treated mice treated with dexamethasone (3 mg/kg every two days, delivered i.p.; 12 mice). All mice in the therapeutic study were given bleomycin sulfate on Day 0 and treated with compounds or vehicle on Days 6–13. On day 14 of both studies, the mice were sacrificed, subjected to bronchoalveolar lavage and the left lung lobe was isolated for histology and the right lung lobe was used for quantification of collagen content. For the histological analysis, the lung lobes were fixed in paraffin and stained with either hematoxylin and eosin or Trichrome. Fibrosis was graded as described by Ashcroft et al. [31] and collagen deposition was scored as outlined by Faress et al. [32]. Measurement of soluble collagen content in lung homogenates was performed according to manufacturer’s instructions using the Sircol Collagen Assay (Biocolor Ltd., Carrickfergus, Northern Ireland).

### Statistics

The data presented are the mean values ± SEM, unless otherwise noted. The statistical differences between the data sets were analyzed by one-way analysis of variance and the Student’s *t*-test was used to determine statistical differences between groups. *P* values <0.05 were considered statistically significant. GraphPad Instat 3 software was used to perform the statistical analysis (GraphPad Software, San Diego, CA).

## Results

### MAP3K19 has a restricted expression pattern

**Fig 1:**
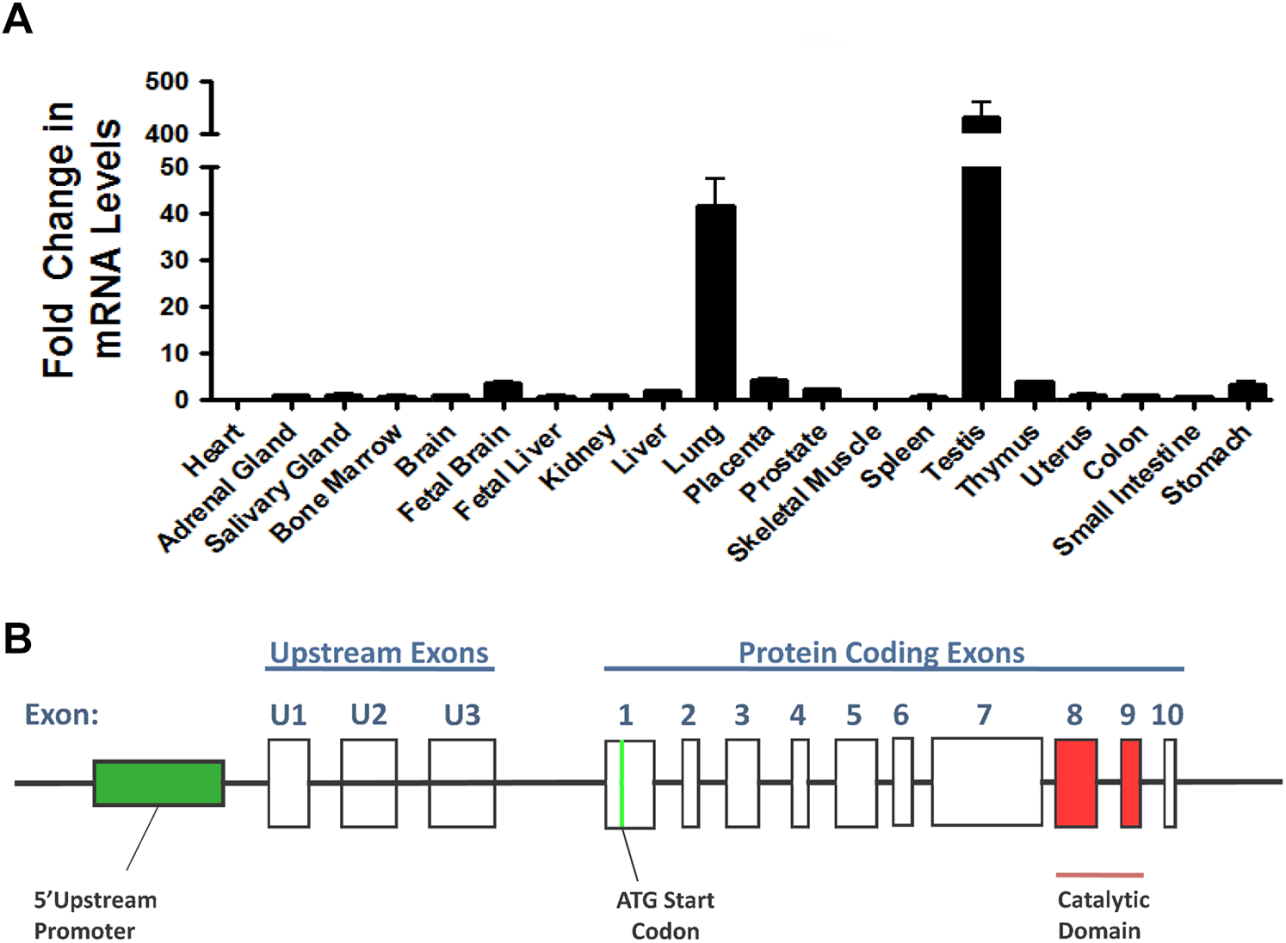
The expression pattern and genomic structure of the MAP3K19 gene. (A) MAP3K19 expression in a panel of twenty human tissues was examined by RT-qPCR and normalized to GAPDH levels, showing the highest level of expression in the lung and testis. The MAP3K19 expression in the kidney was arbitrarily assigned a value of one and fold expression of MAP3K19 in all the other tissues examined were relative to kidney levels. This experiment was repeated twice and the average fold expression is shown ± SEM. The panel of human organ RNA was commercially obtained, and 17 of the 20 RNA samples are pooled from multiple donors (2–62 donors). (B) Deep sequencing of lung specific RNAs (n = 3) coupled with sequence analysis using GENCODE, the Human Genome Consortium HG19 and cufflinks revealed the genomic structure of MAP3K19, located on human chromosome 2. There were three upstream exons, that contain numerous start and stop codons and 10 coding exons. The kinase domain of MAP3K19 is encoded by exons 8 & 9. (C) RT-PCR analysis of MAP3K19 does not show evidence of differential splicing. MAP3K19 exons 1–7 (5’ Exons) and exons 7–10 (3’ Exons) were amplified by RT-PCR, and visualized on an ethidium bromide stained agarose gel, and all positive tissues showed amplified products of the expected size compared to the full length MAP3K19 gene. As a positive RT-PCR control, GAPDH was also amplified from each tissue sample.

MAP3K19 is a large, evolutionary conserved protein composed of 1,328 amino acids (protein accession Q56UN5). Quantitative RT-PCR analysis of MAP3K19 expression in human tissues shows a very limited pattern of expression (Fig 1A). High levels of mRNA expression are only detected lung, and testis, with only a very low level of expression in all other tissue samples examined. Deep sequencing analysis of all lung mRNA transcripts showed no evidence of alternative splicing in adult human lung, as only full length transcripts of MAP3K19 were detected (n = 3 patient samples; G. Hardiman, pers. commun.). Further examination of the deep sequencing analysis showed that MAP3K19 is located on chromosome 2 and is composed of 10 coding exons (labeled exons 1–10 in Fig 1B) and 3 upstream untranslated exons (labeled exons U1, U2 & U3, Fig 1B). There are multiple start and stop codons in the upstream exons U1, U2 and U3. The first start codon leading to a large open reading frame is encoded by exon 1 and a stop codon is in exon 10. Exon 7 is the largest exon, encompassing 2,453 nucleotides, and encodes 2 evolutionary conserved regions of unknown function. The catalytic domain of MAP3K19 is encoded by exons 8 and 9. As RT-qPCR analysis only amplifies an approximately 200 base pair section of the gene, the possibility existed that differential splicing of MAP3K19 may exist in other tissues of the body, and could have gone undetected in our analysis due to the region amplified by RT-qPCR primers. To address this possibility, two sets of PCR primers were designed extending from exon 1 through exon 7 and from exon 7 to exon 10 for semi-quantitative RT-PCR analysis, thereby analyzing the entire protein coding portion of the gene. Consistent with RT-qPCR and deep sequencing findings, we only detected full length transcripts of MAP3K19 using these primer sets, and the expressing tissues were normal lung, COPD lung, trachea, and fetal testis (Fig 1C). All other tissues examined were negative by this analysis. Thus, MAP3K19 appears to have an extremely limited pattern of expression in normal human tissues.

**Fig 2:**
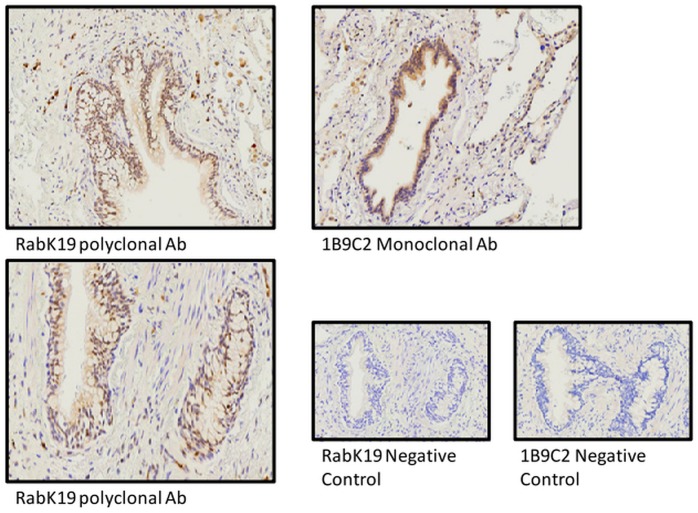
Immunohistochemical analysis showed that MAP3K19 expression in normal lung is predominantly limited to bronchial epithelial cells and interstitial and alveolar macrophage. Normal human lung was stained with either the 1B9C2 anti-MAP3K19 mouse monoclonal antibody (brown staining) or RabK19 rabbit polyclonal antibody (brown staining) and counter-stained with hematoxylin.

**Fig 3:**
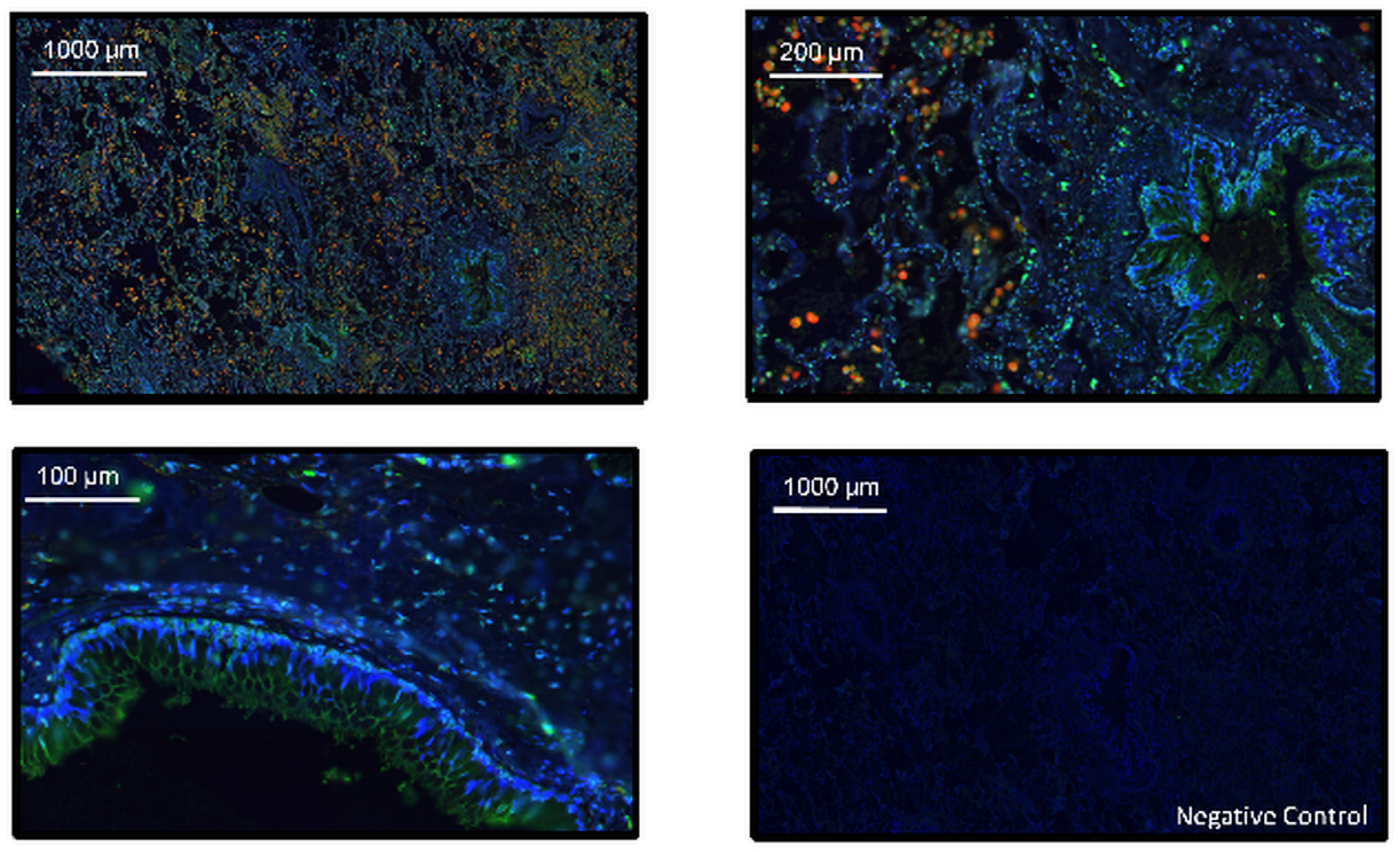
The majority of pulmonary macrophage co-express both MAP3K19 and CD68. Normal human lung was stained for MAP3K19 (Green, RabK19 polyclonal Ab) and CD68 (Red), and counterstained with DAPI.

**Fig 4:**
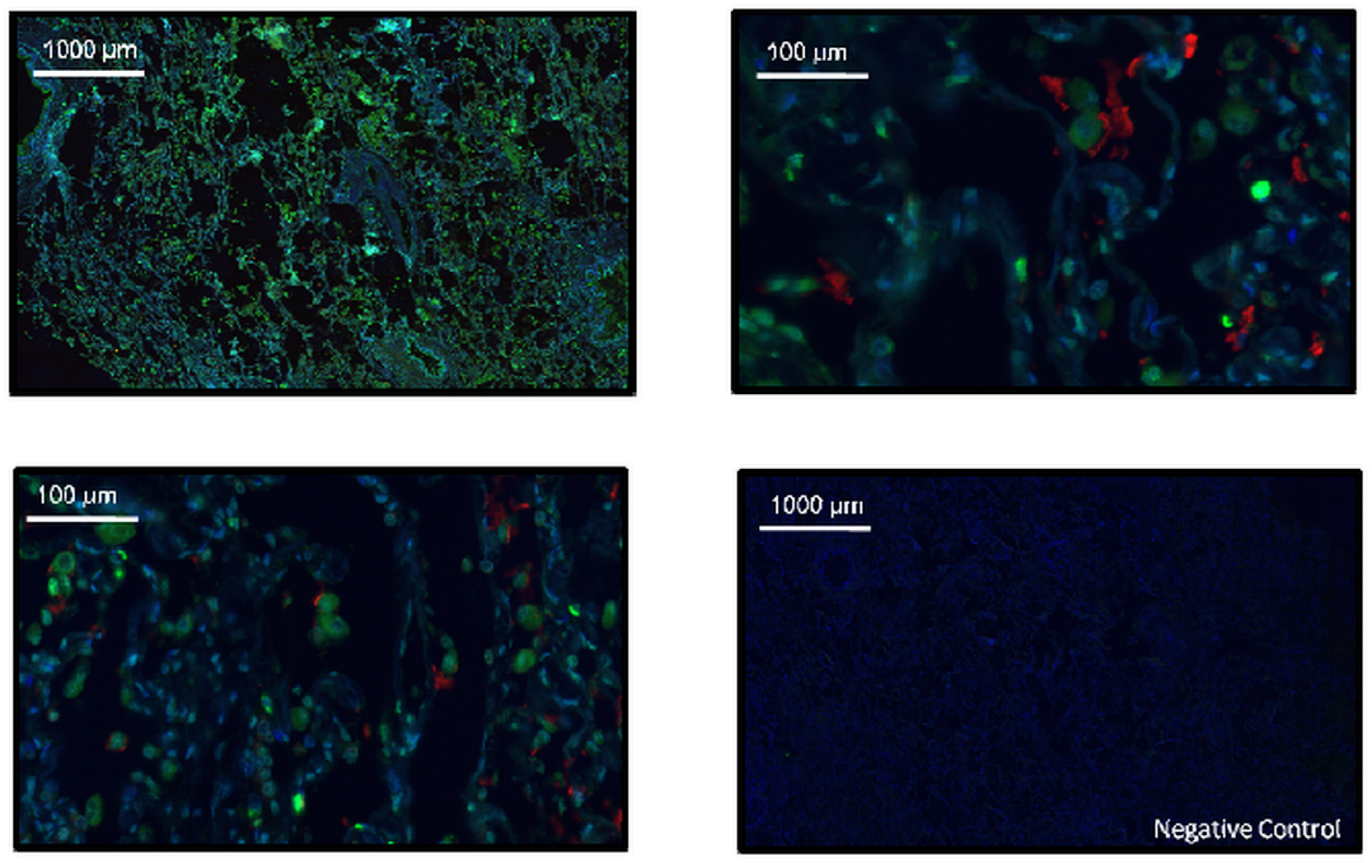
Some type II pneumocytes co-express both MAP3K19 and surfactant protein C. Normal human lung was stained for MAP3K19 (Green, RabK19 polyclonal antibody) and surfactant protein C (Red) and DAPI (Blue).

**Fig 5:**
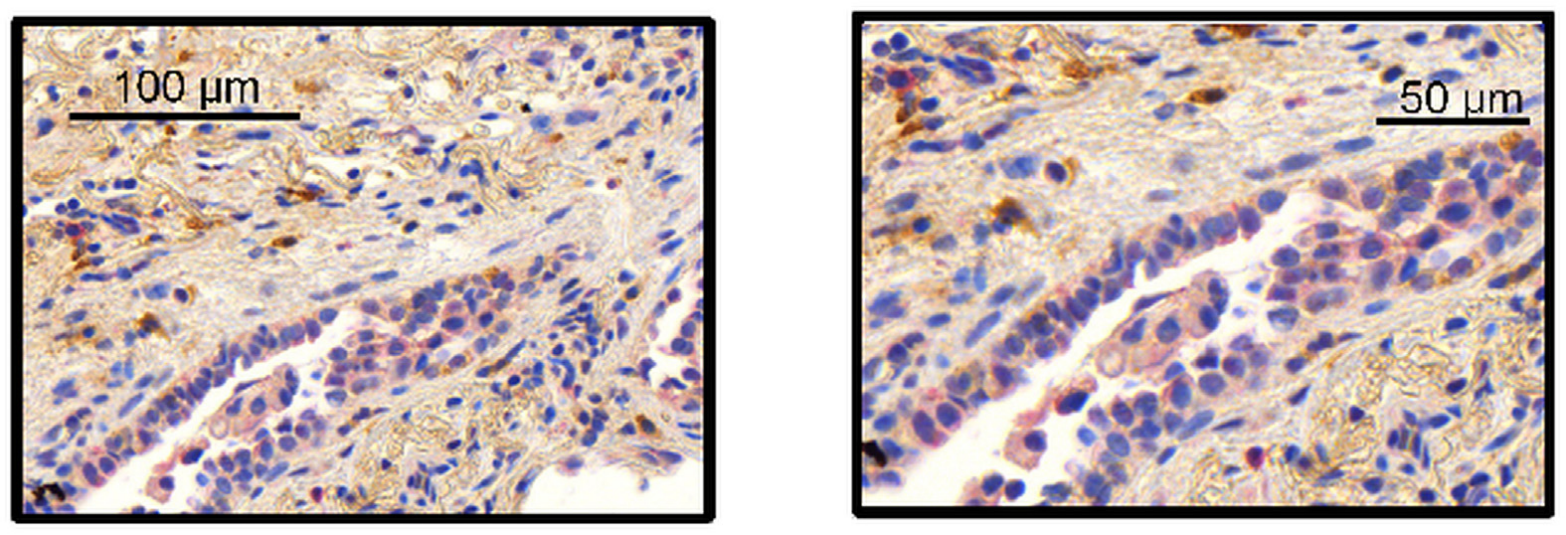
MAP3K19 is expressed in the atypical epithelium tissue that lies adjacent to the fibroblastic foci. MAP3K19 staining of a lung biopsy from a rapid IPF progressor patient showed clear, albeit lower intensity staining, of MAP3K19 (red staining, RabK19 Ab) in the atypical epithelium adjacent to the fibroblastic foci. This staining pattern of the atypical epithelium was found in multiple biopsy sections from IPF patients (n = 3). This section was counter-stained with SSEA-4 (brown stain).

**Fig 6:**
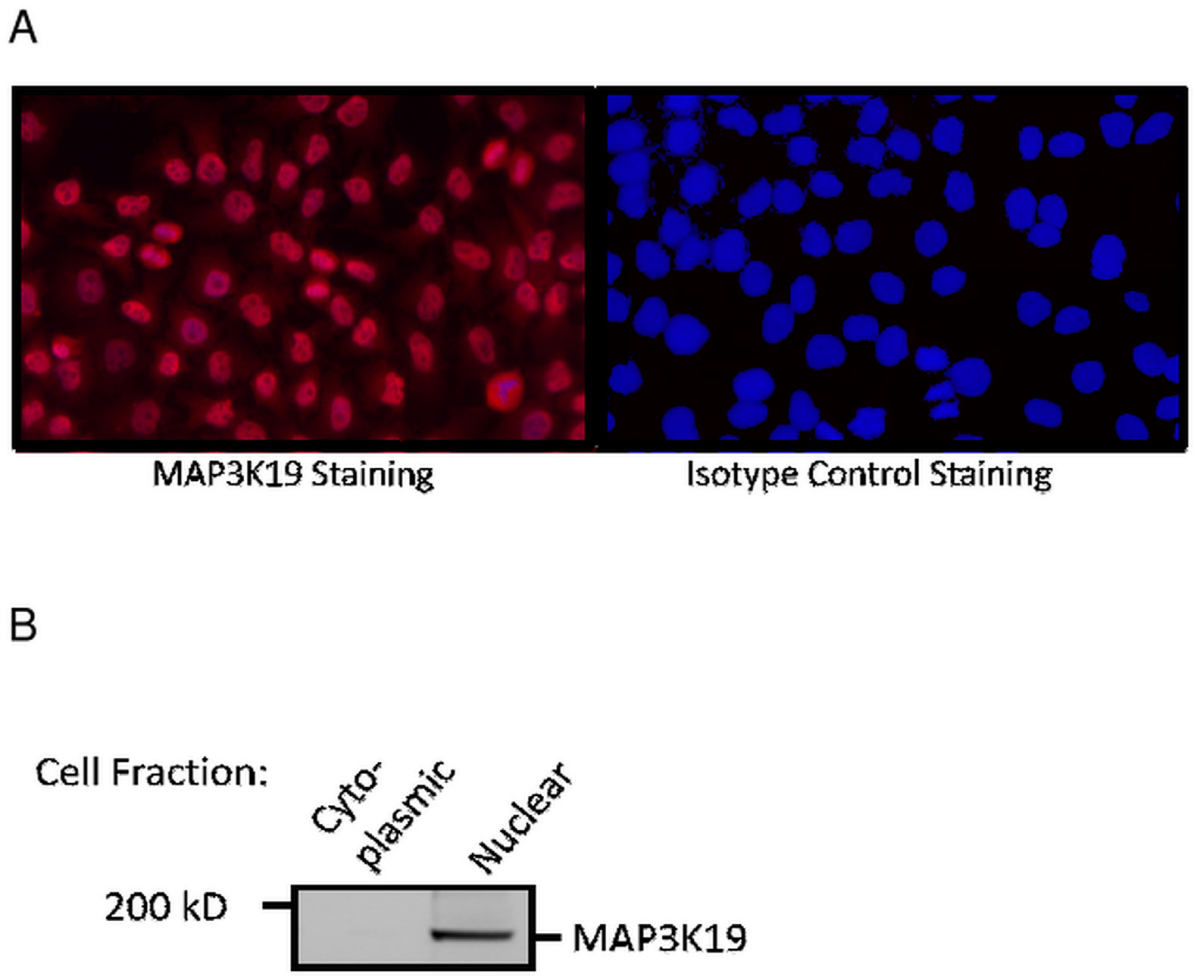
MAP3K19 protein is predominantly localized to the nucleus. (A) HeLa cells were grown on glass cover slips and stained with either MAP3K19 (RabK19 polyclonal antibody) or an isotype control antibody. (B) Western analysis of HeLa cell cytoplasmic or nuclear lysates immunoblotted with anti-MAP3K19 antibody (RabK19) showing nuclear localization of the protein.

Immunostaining analysis of MAP3K19 expression in normal human lung revealed that the kinase is expressed in alveolar and interstitial macrophages, most of whom were CD68^**+**^, neutrophils, which were mainly observed in the blood vessels, randomly distributed fibroblasts, a sub-population of lymphocytes, and some alveolar type II pneumocytes of the epithelium (Figs 2, 3 and 4). This analysis also revealed MAP3K19 expression in the bronchial ciliated epithelial cells of both large and small airways, as well as the trachea. Similar staining results were observed using two different antibodies (1B9C2 and RabK19) directed against different epitopes of MAP3K19. The kinase does not appear to be expressed in cell types surrounding blood vessels in normal human lung tissue. A similar staining pattern was also observed for mouse lungs (S4 Fig). The observation that a subset of macrophages are positive for MAP3K19 expression may also explain the low level of mRNA expression noted in other human tissues by RT-qPCR analysis (Fig 1A). Preliminary analysis of tissue protein arrays by IHC indicates that resident tissue macrophages were positive for MAP3K19 expression (S1 Fig). Further, the IHC tissue array analysis showed that no MAP3K19 protein was detected in the testis, which had the highest level of MAP3K19 mRNA expression per the RT-qPCR analysis, indicating that those mRNA transcripts were not translated into protein (S1 Fig) Histological analysis of lung sections from IPF patients showed that MAP3K19 is additionally expressed by atypical epithelium commonly found adjacent to fibroblastic foci, although the level of staining appeared to be less than in macrophages (Fig 5). A closer analysis of the IHC staining suggested that much of the staining with the MAP3K19 antibodies was both nuclear and cytoplasmic. To investigate this further, HeLa cells stained with a MAP3K19 polyclonal antibody showed a nuclear pattern of protein expression, and this was confirmed by Western blot analysis of different cell fractions (Fig 6).This demonstrated that MAP3K19 can be localized in both the nucleus or the cytoplasm, however in HeLa cells and other cell lines we have examined, the expression appears to be predominantly nuclear. This may represent a dichotomy between in situ cells and tissue culture cells, perhaps due to the growth phase or activation state of the expressing cells.

### MAP3K19 is highly expressed in pulmonary macrophages isolated from human IPF patients

**Fig 7:**
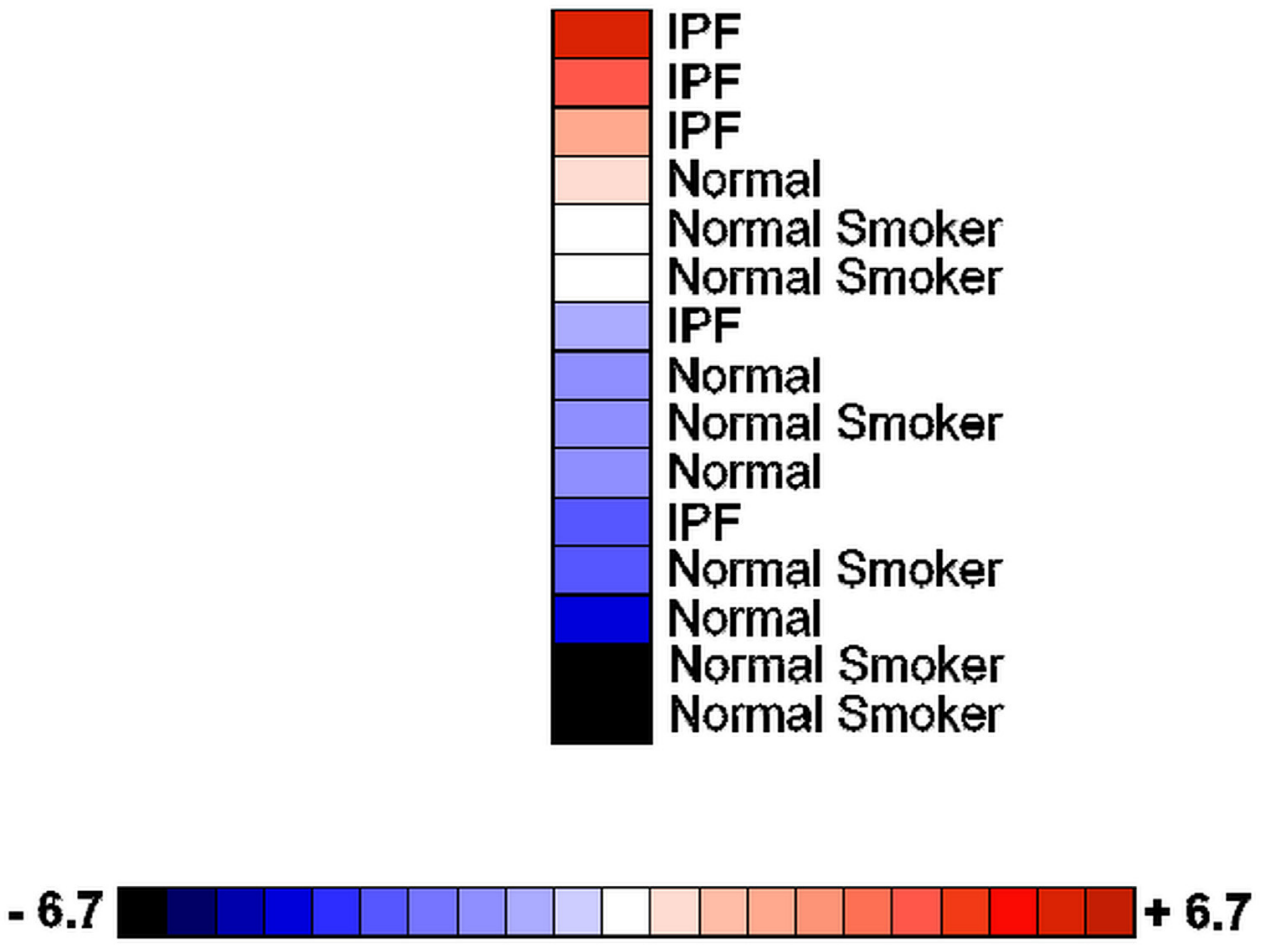
MAP3K19 is over-expressed in bronchoalveolar lavage macrophages from IPF patients. Heatmap showing MAP3K19 expression in BAL macrophages isolated from mild-to-moderate IPF patients (n = 5), normal controls (n = 4) and healthy smokers (n = 6) as determined by RT-qPCR analysis. The data are normalized to GAPDH expression and the fold changes are depicted on the color scale. The RT-qPCR is representative of two independent experiments (biological replicates) performed in duplicate. Anova analysis of MAP3K19 expression levels among the different groups revealed that the difference in mRNA levels between the IPF group, with the lowest expressing patient omitted, and either the normal patients or smokers was significant, P<0.01.

As MAP3K19 is expressed in macrophages, we wanted to determine whether the gene is over-expressed in the pulmonary macrophages of IPF patients. RNA obtained from macrophages purified from bronchoalveolar lavage of normal patients, smokers and IPF patients was subjected to RT-qPCR analysis. This revealed that IPF patients had increased levels of MAP3K19 expression in BAL macrophages compared to non-diseased patients (Fig 7). Taken together with the IHC staining of IPF lungs, these observations strongly suggested that MAP3K19 is upregulated in the lungs of IPF patients.

### Inhibition of MAP3K19 blocks TGF-β-induced R-Smad nuclear translocation

**Fig 8:**
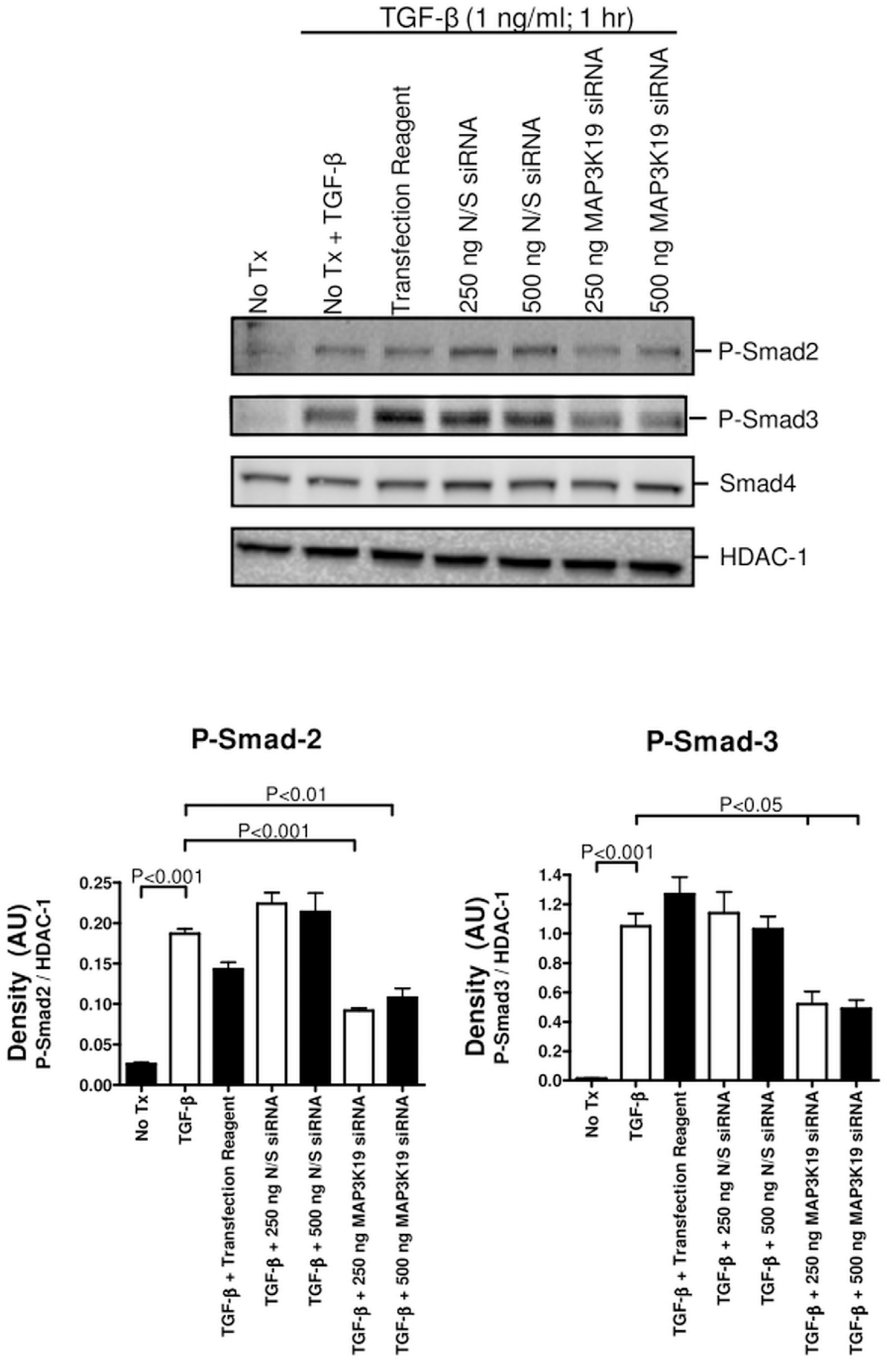
MAP3K19 promoted the nuclear accumulation of the activated R-Smads phospho-Smad2 and phospho-Smad3 following TGF-β1 stimulation. (A) HeLa cells were transfected with nonsense siRNA (250 or 500 ng) or MAP3K19 siRNA (250 or 500 ng). Eighteen hours later, the cells were treated with TGF-β1 (1 ng/ml) for 1 hour, harvested and nuclear extracts were isolated. Nuclear lysates were examined by Western analysis for P-Smad2, P-Smad3 and Smad4 levels, and the blots were re-probed with HDAC-1 to show equal protein loading. Densitometry was performed comparing either the P-Smad2/HDAC-1 levels or the P-Smad-3/HDAC-1 levels. Smad4 levels were not examined by densitometry, as they were not affected. This experiment was repeated three independent times and a representative experiment is shown.

As TGF-β is thought to play a central role in the development of pulmonary fibrosis and MAP3K19 is both highly expressed in BAL macrophages from IPF patients and in epithelial cells adjacent to fibrotic foci, we next examined whether inhibition of MAP3K19 kinase activity would affect TGF-β signal transduction. We therefore transfected anti-MAP3K19 siRNA into HeLa cells, and noticed a knock-down in MAP3K19 levels in the MAP3K19 siRNA treated cells compared to N/S siRNA or mock transfected cells, as detected by RT-qPCR (S2 Fig). Following TGF-β1 stimulation, we observed decreased nuclear phospho-Smad2 and phospho-Smad3 levels (Fig 8). Transfection of a kinase-dead mutant of MAP3K19 (K1089R) into either HeLa or A549 cells also resulted in a decrease of phospho-Smad2 and Smad3 nuclear levels upon TGF-β1 treatment, suggesting that MAP3K19 promoted TGF-β signaling (data not shown, d.n.s.).

**Fig 9:**
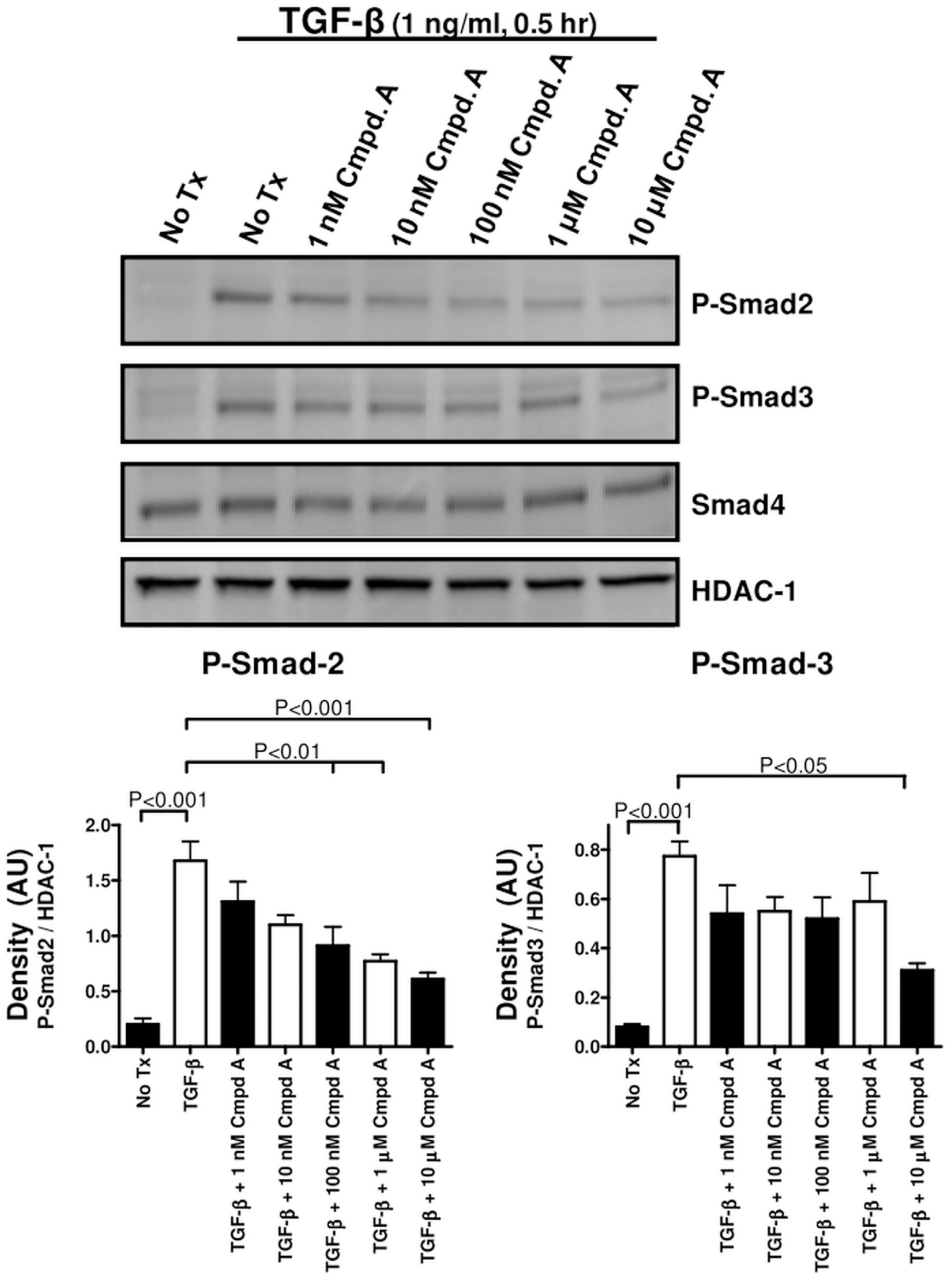
Inhibition of MAP3K19 by Compound A can abrogate the nuclear localization of R-Smads following TGF-β1 stimulation in a dose-dependent manner. THP-1 cells were treated for 18 hours with 150 nM PMA and then cultured in fresh medium overnight to promote M0 macrophage differentiation. At that point, the cells were pre-treated with the indicated concentrations of Compound A, a highly specific and potent small molecule antagonist of MAP3K19 kinase activity, for 30 minutes. The cells were then treated with TGF-β1 (1 ng/ml) for an additional 30 minutes, harvested, and nuclear lysates were made. Western blot analysis showed a dose-dependent decrease in nuclear phospho-Smad2/3 levels, while Smad4 levels were unaffected in this time course. As a loading control, blots were rehybridized with anti-HDAC-1 antibody. Similar results were observed with the A549 and HeLa cell lines and this blot is representative of n = 3 experiments. Densitometric analysis of P-Smad2/HDAC-1 levels between the different samples and P-Smad3/HDAC-1 is also shown.

**Fig 10:**
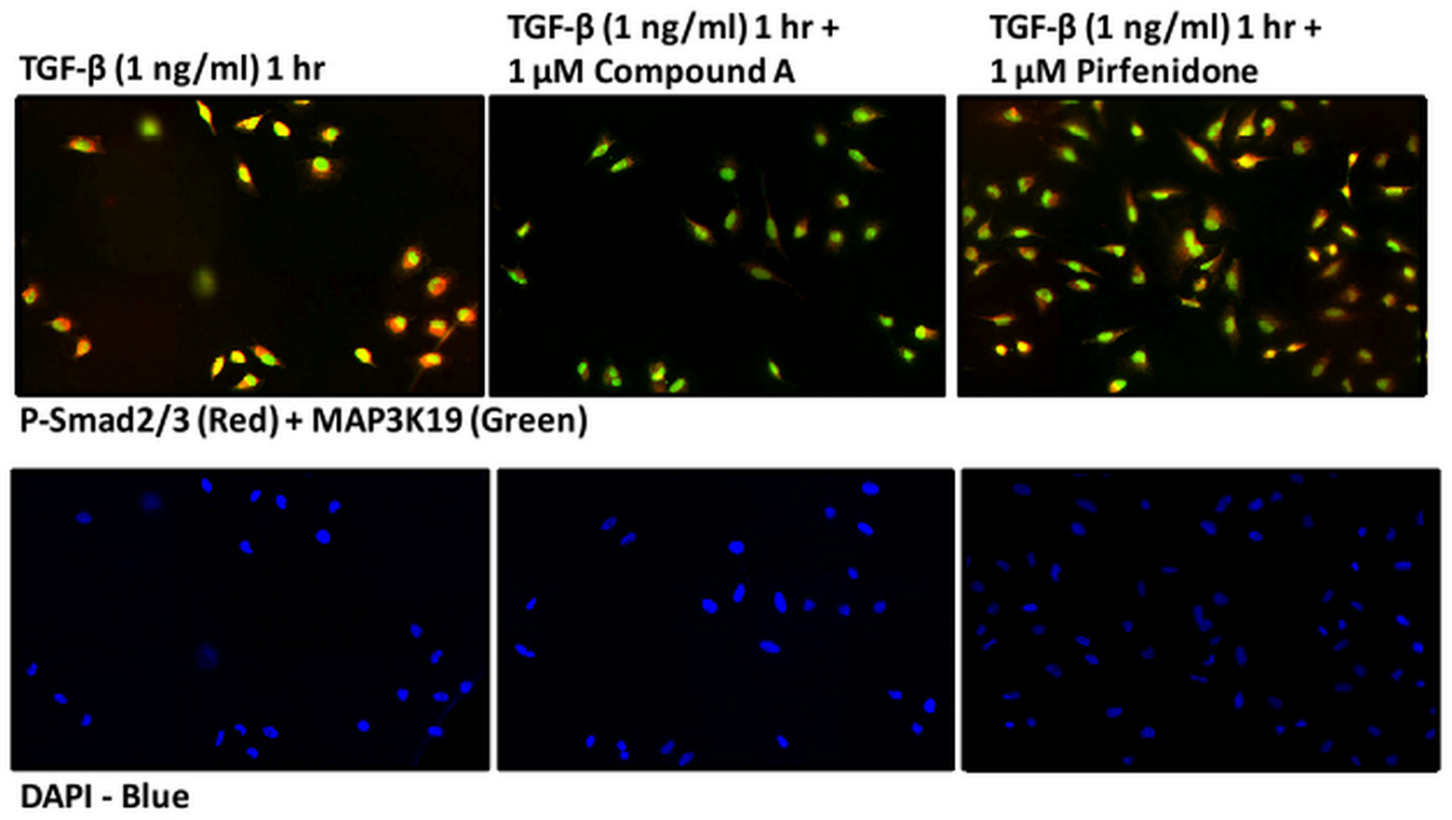
The MAP3K19 inhibitor, Compound A, can block phosphorylated R-Smad nuclear accumulation, whereas pirfenidone has a negligible effect, following TGF-β1 stimulation. HeLa cells were cultured overnight on glass cover slips and pretreated with vehicle (DMSO), Compound A (1 μM) or pirfenidone (1 μM) for 30 minutes and treated with TGF-β1 (1 ng/ml) for one hour, prior to fixation and staining with anti-MAP3K19 (green staining, RabK19 polyclonal antibody) and anti-phospho-Smad2/3 (red staining). The cells were counterstained with DAPI nuclear stain. This experiment was repeated two independent times, and a representative experiment is shown. Similar results were observed with the A549 lung epithelial cell line.

To further examine this observation, we identified several novel, small molecule inhibitors of the MAP3K19 kinase activity that are both highly selective and potent. Western analysis of PMA-differentiated THP-1 monocytic cells treated with a potent, highly selective, small molecule MAP3K19 inhibitor, Compound A, showed a dose-dependent decrease in the amount of phospho-Smad2 in the nucleus following TGF-β1 treatment (Fig 9). Phospho-Smad3 levels were also decreased, although to a lesser extent than phospho-Smad2, and levels of the common-mediator Smad, Smad-4 were not affected. Similar results were observed when HeLa cells were examined histologically. Treatment of the cells with TGF-β1 resulted in the nuclear translocation and accumulation of the receptor Smads, as detected by a phospho-Smad 2 and 3 dual specific antibody (Fig 10). However, when cells were also treated with Compound A, the amount of phospho-Smad2/3 positive cells was greatly reduced. Interestingly, there were no increased levels of cytoplasmic phospho-Smad2/3 either, indicating that MAP3K19 inhibition blocks the total cellular accumulation of the phosphorylated receptor Smads (Fig 10). Further, treatment of cells with 1 μM of pirfenidone had a negligible effect on phospho-Smad2/3 nuclear accumulation. Similar results were observed in both A549 lung epithelial carcinoma cells for both Western and IHC analysis. Using either siRNA or a specific inhibitor, the effect of MAP3K19 inhibition on TGF-β signaling and decreased nucleocytoplasmic shuttling was consistent.

**Fig 11:**
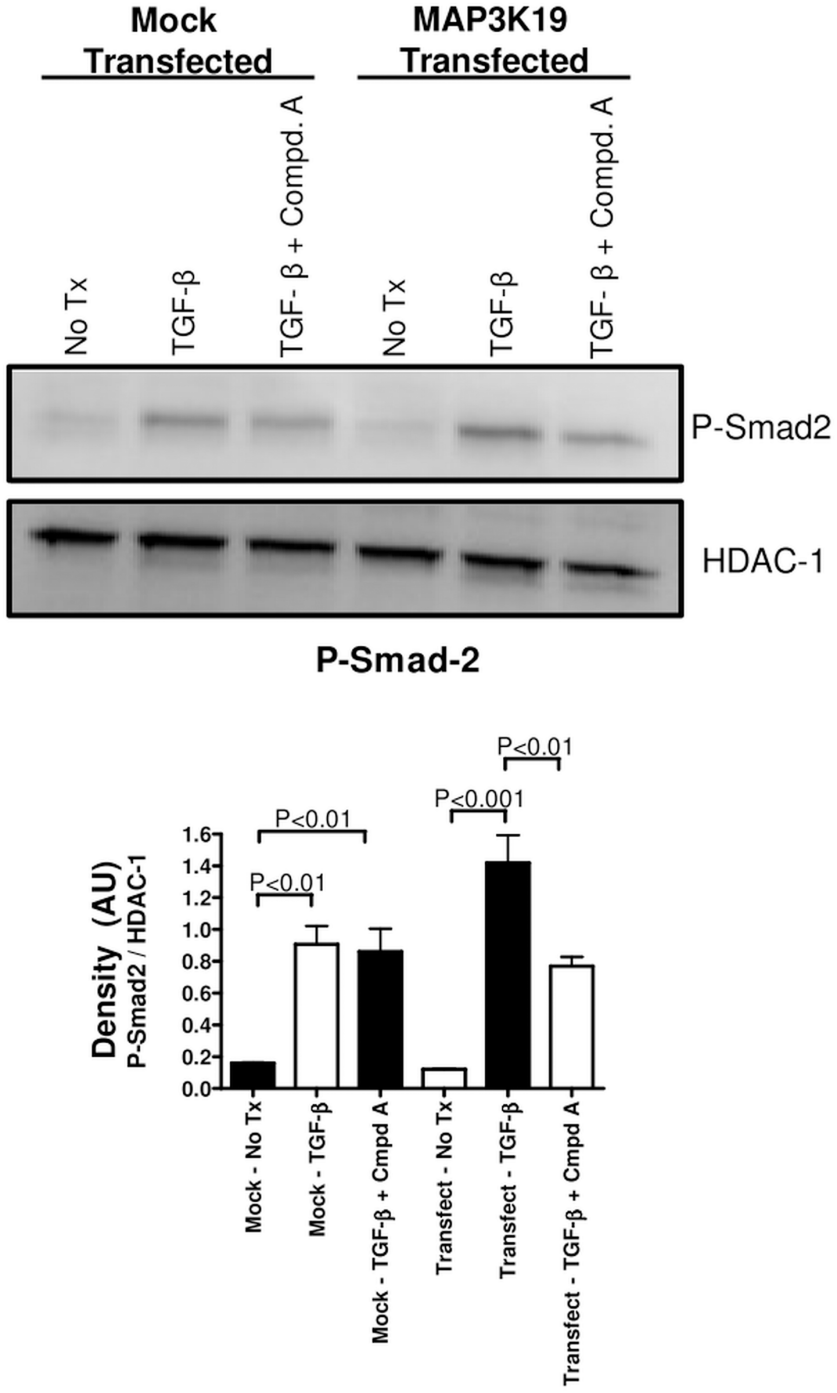
Transfection of MAP3K19 renders HEK cells susceptible to Compound A-mediated inhibition of P-Smad2 nuclear translocation. HEK cells, which express very low levels of MAP3K19 as determined by RT-qPCR analysis, were transfected with either empty vector (pcDNA) or MAP3K19. Following an overnight culture, the cells were pretreated with Compound A (1 μM) for 30 minutes and treated with TGF-β1 (1 ng/ml) for one hour. The cells were then harvested, and the nuclear lysates were assayed by Western analysis for nuclear phospho-Smad2 localization. The blot was then re-hybridized with HDAC-1 to show equal protein loading. Densitometric analysis of P-Smad2/HDAC-1 levels from the Western blot is shown. This experiment is representative of two independent experiments.

Transfection of HEK293 cells, which express very low endogenous levels of MAP3K19 mRNA, with a MAP3K19 expression plasmid have slightly increased levels of nuclear phospho-Smad2, compared to empty vector transfected cells upon TGF-β1treatment. Administration of the MAP3K19 antagonist Compound A (1 μM), decreased nuclear phospho-Smad2 levels in the MAP3K19 transfected cells to much a greater degree than mock transfected cells, again implicating a role for MAP3K19 in regulating nuclear levels of Smad2 when MAP3K19 is present in cells (Fig 11 and S3 Fig). Taken together, these results show that MAP3K19 can regulate the TGF-β signaling pathway, and that inhibition of MAP3K19 strongly reduced the nuclear localization of the R-Smads.

### Effect of MAP3K19 on TGF-β-induced gene transcription

**Fig 12:**
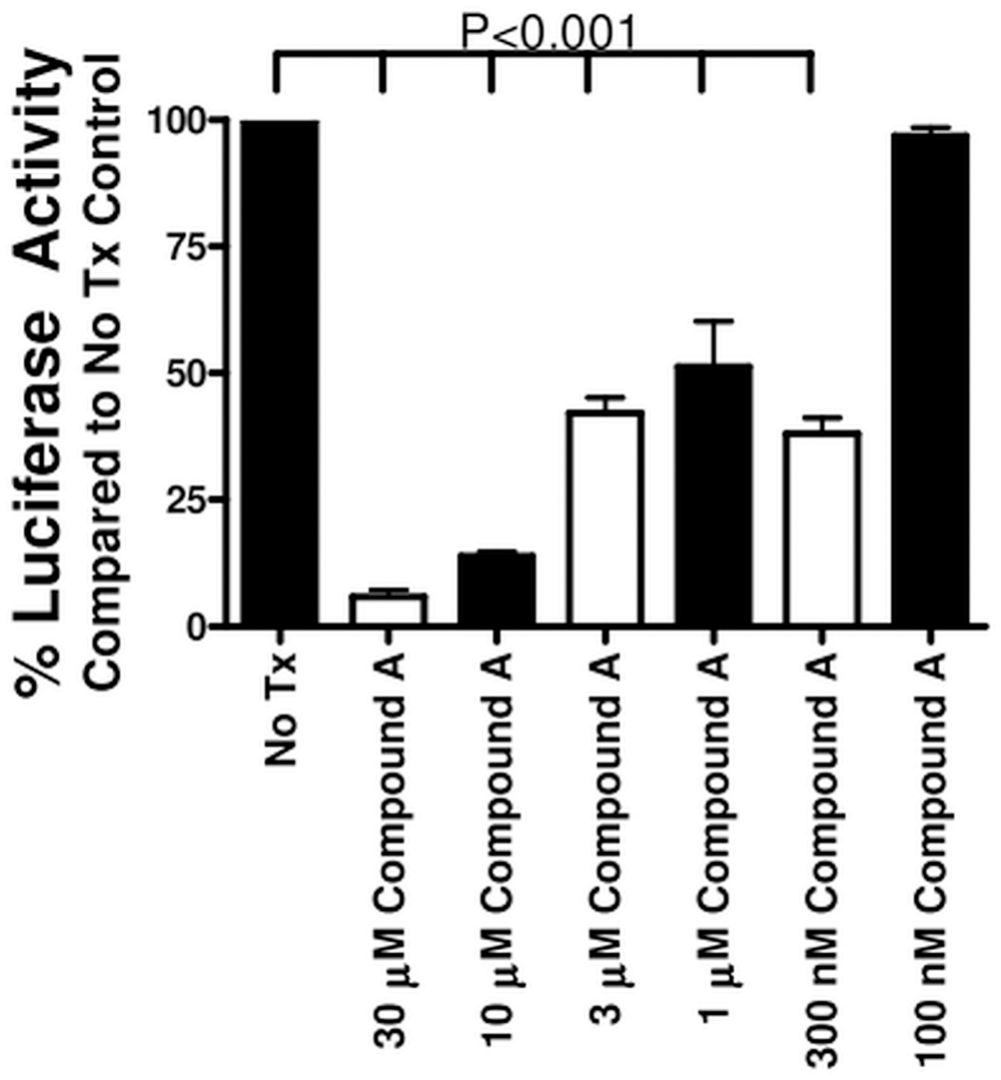
Inhibition of MAP3K19 kinase activity by Compound A inhibits Smad2/3-mediated transcription. A549 cells were stably transfected with a construct containing the Smad binding element (SBE) linked to a luciferase reporter gene. The cells were plated out in 96-well plates, incubated with the designated concentrations of Compound A, and treated with 0.1 ng/ml of TGF-β1 for 18 hours. At that point, the cells were assayed for luciferase activity, indicative of Smad 2/3 transcriptional activation. Wells that received no Compound A, only TGF-β1, were normalized to 100% luciferase activity. The mean percentage of luciferase activity is shown ± SEM. A representative experiment is shown (n = 3).

**Fig 13:**
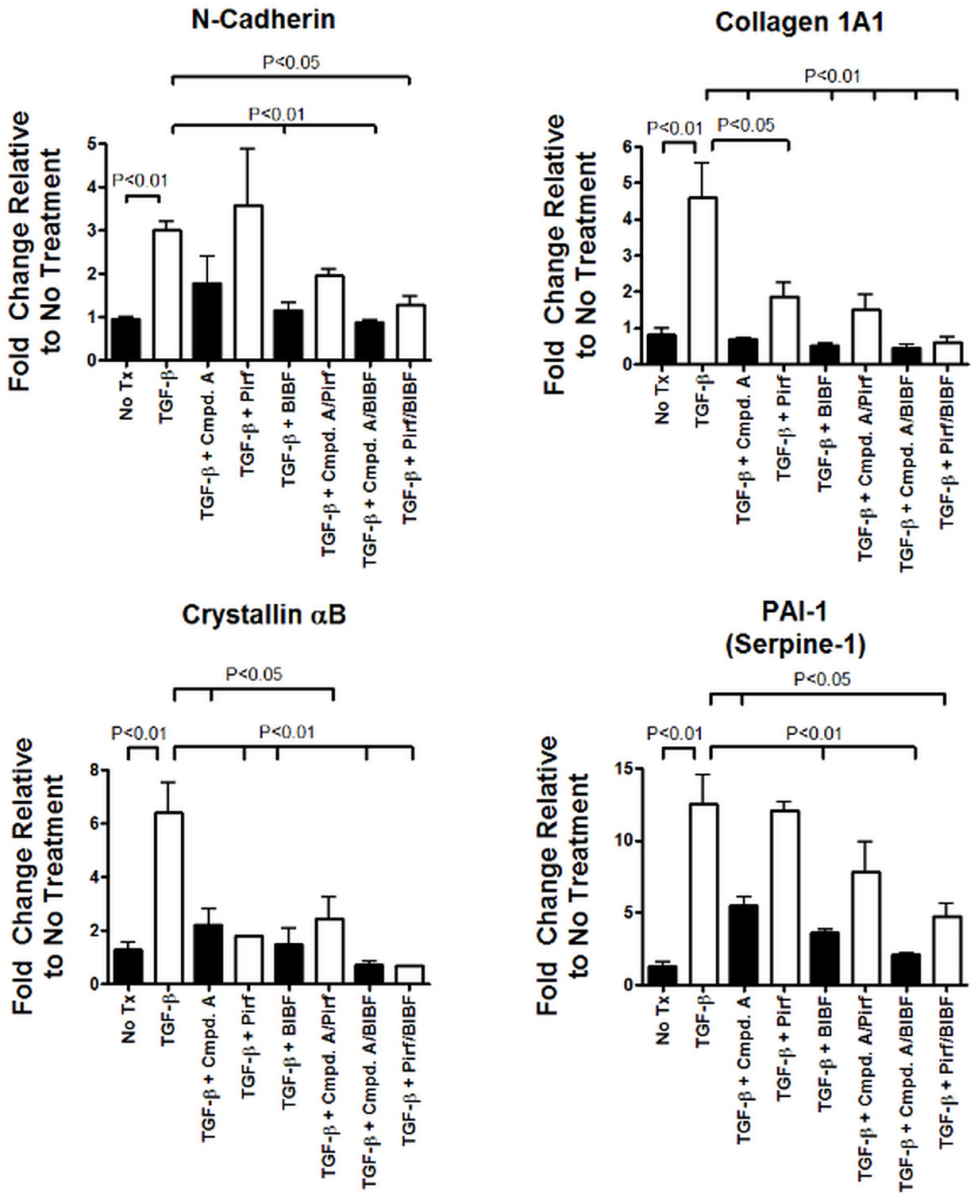
Inhibition of MAP3K19 blocks TGF-β1-mediated gene transcription. A549 lung epithelial cells were treated with 1 μM of Compound A, pirfenidone or nintedanib, individually or in combination, and TGF-β1 (1 ng/ml) for 12 hrs. RNA was harvested and relative mRNA transcript levels were measured by RT-qPCR. Results are shown for (A) collagen 1A1, (B) N-cadherin, (C) PAI-1 and (D) αB-crystallin. All samples were normalized to GAPDH, and results are expressed as fold change compared to untreated (No Tx) samples. The RT-qPCR was run in duplicate samples and the mean ± SEMs shown. This experiment was repeated three independent times with similar results and a representative experiment is shown. Similar results were obtained for HeLa cells and Beas-2B cells.

Having established that inhibition of MAP3K19 kinase activity can block R-Smad nuclear translocation following TGF-β stimulation, we wanted to examine what effect this had on TGF-β-induced gene transcription. To address this question, we first made stable A549 cell transfectants containing a Smad binding element (SBE) linked to a luciferase reporter gene. The cells were treated with different doses of Compound A and 0.1 ng/ml of TGF-β for 18 hours. The luciferase activity was then determined, and showed that Compound A decreased Smad-dependent transcriptional activity in a dose-related manner (Fig 12). We next investigated the effect of MAP3K19 inhibition directly on TGF-β-induced genes by RT-qPCR analysis of A549 cells. Pirfenidone and nintedanib were also included in this analysis with Compound A (all treatments at 1 μM), either alone or used in combination. As shown in Fig 13, Compound A by itself reduced mRNA levels of collagen 1A1, N-cadherin, PAI-1 (Serpin-1) and αB-crystallin, similar to nintedanib. Pirfenidone, used singularly, only reduced collagen 1A1 levels and αB-crystallin, but not N-cadherin or PAI-1 levels. Interestingly, Compound A appeared to synergize with either pirfenidone or nintedanib when used in combination to reduce TGF-β-dependent gene transcription, perhaps suggesting a different mechanism of action.

### MAP3K19 inhibitor reduced TGF-β-induced protein levels of PAI-1 and acted additively with pirfenidone

**Fig 14:**
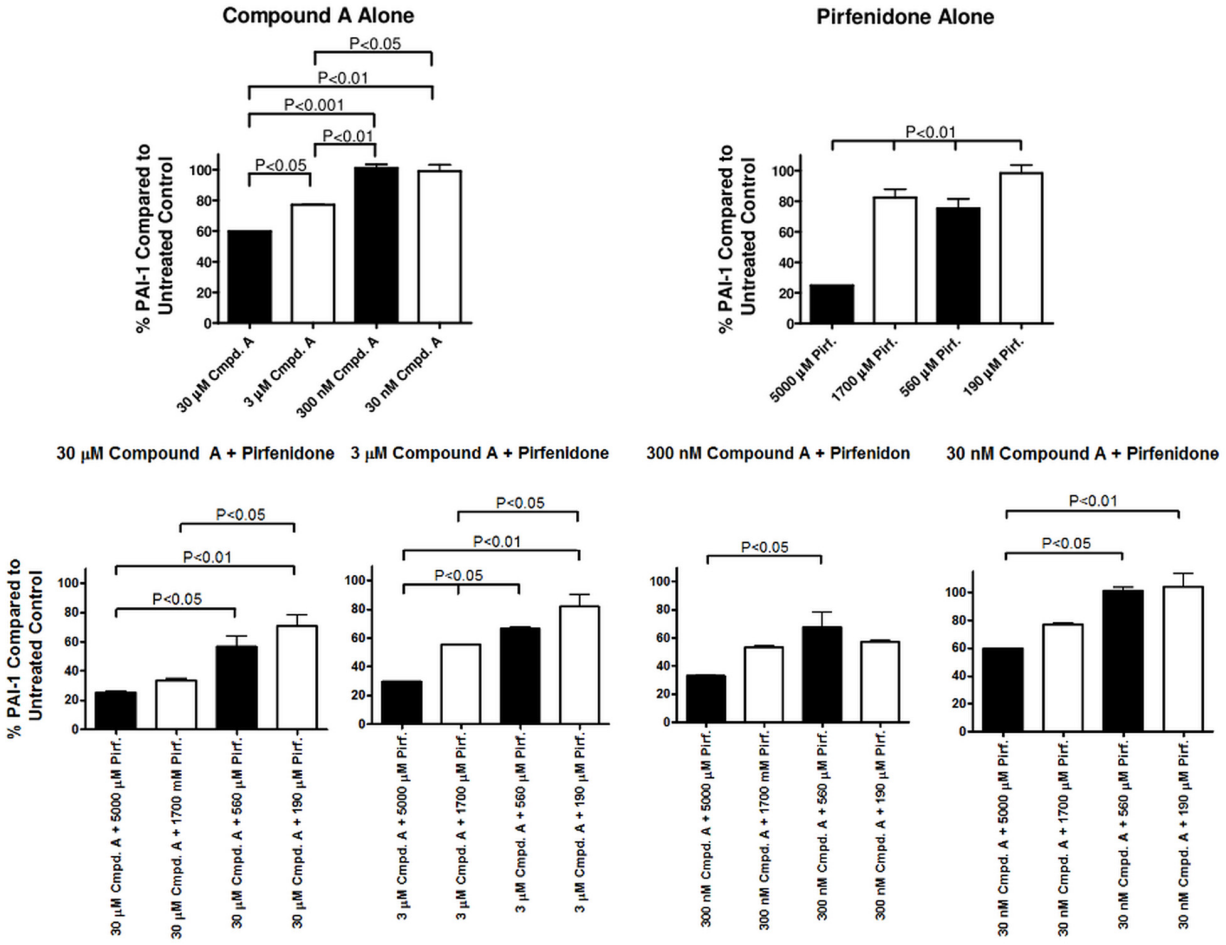
MAP3K19 antagonist inhibited TGF-β1-induced protein and acted additively with therapeutic doses of pirfenidone. A549 cells were treated with 30 μM, 3 μM, 300 nM or 30 nM of Compound A or 5000 μM, 1700 μM, 560 μM or 190 μM of pirfenidone alone or in combination, and TGF-β1 (1 ng/ml) for 24 hrs. Cell lysates were isolated and PAI-1 (Serpin-1) protein levels were determined by ELISA. Results shown were the percentage of PAI-1 protein compared to levels from cells treated only with TGF-β1. Results are representative of two independent experiments.

Using the lung epithelial cell line A549 as a model, we next investigated the effect of Compound A on PAI-1 protein levels either alone or in combination with various therapeutically relevant doses of pirfenidone (Fig 14). Both 30 μM and 3 μM concentrations of Compound A significantly reduced PAI-1 protein levels in response to TGF-β treatment, whereas 300 nM and 30 nM concentrations had a minimal effect. Pirfenidone, when administered at 5000 μM reduced PAI-1 levels by almost 80%, but 1700 μM and 560 μM of pirfenidone reduced PAI-1 levels to a lesser degree. Pirfenidone given at 190 μM had no effect PAI-1 protein levels. When pirfenidone was given together with Compound A, there was a negligible additive effect on PAI-1 levels detected at the 5000 μM dose of pirfenidone. However, at the lower doses of pirfenidone tested, the addition of Compound A reduced PAI-1 protein levels in a dose-dependent manner. We did not detect any toxicity or decreased viability of A549 cells with any concentration of either Compound A or pirfenidone used. These results confirmed and extended the observations seen at the mRNA level illustrating that Compound A and pirfenidone appear to have an additive effect in reducing TGF-β-induced gene transcription and protein production.

### Inhibition of MAP3K19 attenuated bleomycin-induced pulmonary fibrosis

**Fig 15:**
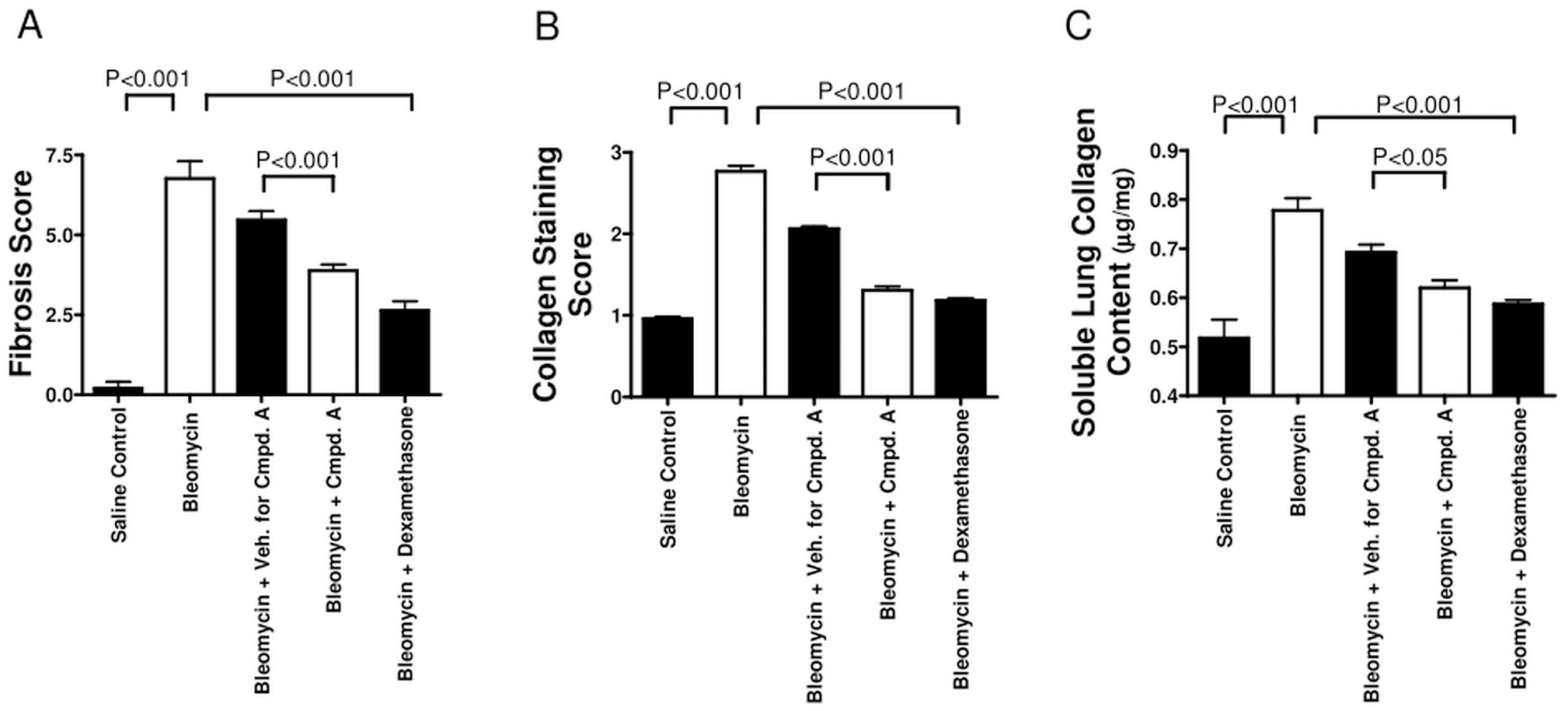
MAP3K19 inhibition protected mice from bleomycin-induced pulmonary fibrosis when administered prophylactically (A-C). C57Bl/6J mice had saline (n = 5 mice) or bleomycin instilled intra-tracheally (i.t.) on Day 0 and for the prophylactic treatment, received either the vehicle control (n = 12) or Compound A (10 mg/kg; n = 12) delivered daily orally, dexamethasone administered intra-peritoneally (i.p.) every other day (3 mg/kg, n = 12), or no treatment (n = 5). On day 14, the mice were sacrificed, and histological criteria were used to assess (A) the pulmonary fibrosis determined using the Ashcroft scale [31], (B) the collagen deposition was scored using the method described by Faress et al. [32], and (C) the soluble collagen content in lung homogenates was determined using a Sircol assay. Values shown are the mean ± SEM.

**Fig 16:**
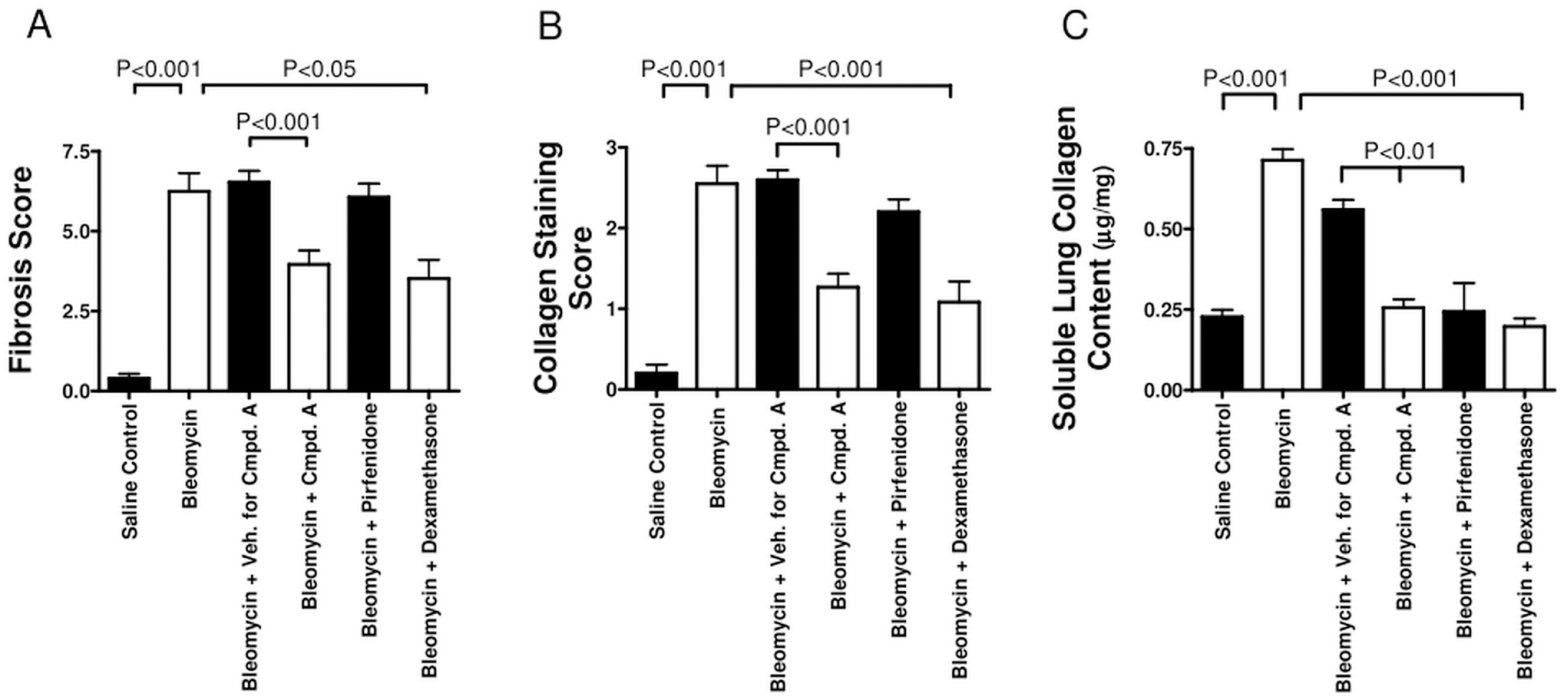
Therapeutic dosing of Compound A protected mice from bleomycin-induced pulmonary fibrosis. For the therapeutic arm of this experiment, the cohorts were (1) i.t.-delivered saline control group (n = 5 mice), (2) bleomycin-instilled no treatment group (n = 5), (3) bleomycin-treated vehicle control cohort (n = 12), (4) bleomycin-instilled Compound A treated (10 mg/kg) group (n = 12), (5) bleomycin-instilled pirfenidone treated (10 mg/kg) cohort (n = 12), and (6) bleomycin-instilled group with dexamethasone (3 mg/kg delivered i.p.) administered every other day (n = 12). Treatment for mice that received bleomycin started on day 6 and continued to day 13. As with the prophylactic study, mice were scored for fibrosis (A), collagen deposition (B) and the soluble lung collagen content (C). The mean scores ± SEM are shown.

**Fig 17:**
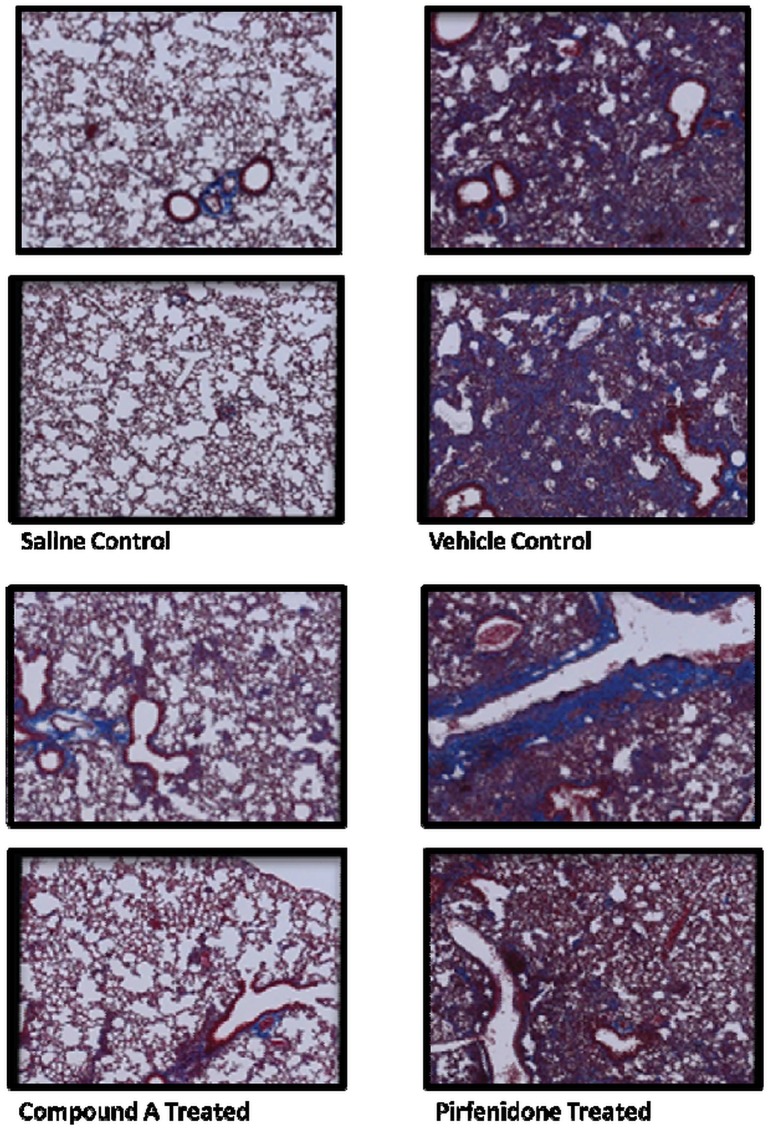
Histological analysis of the lungs from bleomycin-treated mice in the presence or absence of Compound A or pirfenidone treatment. Representative histology from Masson’s Trichrome stained sections of saline-instilled mice, vehicle treated, Compound A treated and pirfenidone treated mice are shown in panel (G). MAP3K19 staining of bleomycin treated mouse lungs, from a separate experiment, is shown in the Supplementary Data (S4 Fig).

Given that MAP3K19 is expressed by multiple pro-inflammatory cell types in the lung and that it can modulate TGF-β-mediated signal transduction and gene transcription, we wanted to ask whether inhibition of MAP3K19 could influence the development of pulmonary fibrosis in vivo, using a murine model of bleomycin-induced pulmonary fibrosis. In the first experiment, mice were dosed prophylactically every day with either the MAP3K19 inhibitor Compound A (delivered orally), a drug vehicle control solution or dexamethasone (delivered i.p. every second day of the experiment). Mice treated with the MAP3K19 inhibitor or dexamethasone had significantly reduced pulmonary fibrosis (Fig 15A), collagen deposition (Fig 15B) and lung collagen content (Fig 15C). The decrease in fibrosis and collagen deposition due to MAP3K19 inhibition is also readily observed in the histological sections obtained from the mice. We next wanted to investigate whether MAP3K19 inhibition could act therapeutically in this model of pulmonary fibrosis. We also included in this study pirfenidone, which is the standard of care for many IPF patients. Mice were intra-tracheally instilled with the bleomycin on day 0 and on day 6 the compounds were administered daily, except for dexamethasone, which was given every second day until the completion of the study on day 13. Similar to the first study, therapeutic dosing of a MAP3K19 inhibitor and dexamethasone significantly reduced fibrosis and collagen deposition, whereas pirfenidone had little to no effect on these readouts (Fig 16A and 16B). Pulmonary collagen content was significantly decreased for all three drugs compared to vehicle control, and the bleomycin control (Fig 16C). The histological analysis shows a similar positive effect of the MAP3K19 compound, particularly compared to pirfenidone (Fig 17). Taken together, these results demonstrate that inhibition of MAP3K19 significantly attenuated the development of pulmonary fibrosis in a murine bleomycin model, and these effects may be attributable, at least in part, to inhibition of the TGF-β pathway.

## Discussion

The experiments reported here used the cell lines A549, a human lung-derived epithelial carcinoma cell line, THP-1 cells, a human leukemic monocytic cell line, and HeLa, a human cervical carcinoma-derived epithelial cell line. These cells were used because of their MAP3K19 expression. The nontransformed primary pulmonary-derived cells examined did not express MAP3K19 unless induced by cellular stress, similar to HEK cells. Thus, they did not represent viable experimental alternatives aimed at discovering the biological role of MAP3K19. The cells used also responded in a physiologic manner to TGF-β1 stimulation. These two characteristics made the cell lines utilized in this study viable experimental alternatives.

TGF-β is a potent profibrogenic cytokine that plays a central role in the development of fibrosis by modulating fibroblast proliferation and chemotaxis, stimulating the production and deposition of connective tissue, and by inhibiting connective tissue breakdown. Our results show that TGF-β signaling and downstream gene transcription is promoted by MAP3K19 regulated nucleocytoplasmic shuttling of the activated R-Smads, in cells which express MAP3K19. The mechanism by which MAP3K19 accomplished this, and which proteins it interacted with is still under examination. Nonetheless, inhibition of MAP3K19 activity by either a small molecule or siRNA resulted in decreased phospho-Smad2/3 nuclear accumulation (Figs 8, 9 and 10). In our studies of TGF-β signaling by Western analysis or IHC, we did not detectphospho-Smad2 in the cytoplasm upon inhibition of MAP3K19. Additionally, biochemical studies using 1 μM of Compound A have shown that the antagonist compound did not inhibit the TβRI kinase or ALK5. These results suggest that Compound A is involved in the inactivation of phospho-Smad2/3 and subsequent nuclear export. This could occur by a MAP3K19-mediated activation of a phosphatase, such as PPM1A, which has been shown to dephosphorylate and inactivate Smad2 and Smad3 [33, 34]. Alternatively, Smad2/3 are also negatively regulated by the ubiquitin ligase Nedd4L, which targets the activated R-Smads for proteosome-mediated degradation [35]. This process could also be influenced by MAP3K19. Preliminary evidence has shown an increase in cytoplasmic total Smad2 levels following Compound A addition, suggesting a phosphatase-mediated event, however these hypotheses are presently under further investigation.

In examining TGF-β-induced genes, we found a Compound A-dependent decrease in RNA levels. Specifically, two genes involved in fibrosis and EMT, collagen 1A1 and N-cadherin, are down-regulated, as was the protease inhibitor PAI-1 and the chaperone molecule αB-crystallin (Fig 13) [36–38]. Our in vitro experiments revealed that at similar concentrations, Compound A seems as potent as nintedanib in the inhibition of TGF-β-induced gene expression. Pirfenidone was less potent at these doses. This was also observed in vivo, where either Compound A or pirfenidone were administered at 10 mg/kg (Figs 16 and 17). Compound A was much more effective at blocking fibrosis and inflammation. In vitro experiments did show, however, that Compound A administered with either pirfenidone or nintedanib did appear to act synergistically at the level of RNA or PAI-1 protein expression (Figs 13 and 14).

Numerous studies have shown that blocking TGF-β signaling could have a beneficial therapeutic effect on pulmonary fibrosis in both animal models and in the clinic. For instance, a small molecule inhibitor of TβRI, or ALK5, has been shown to reduce pulmonary fibrosis induced by adenovirus-mediated gene transfer of TGF-β [39]. However, numerous ALK5 inhibitors are currently in development or clinical trials and toxicity and side effects are common with drugs against this kinase [40]. Smad3 null mice were protected against fibrosis caused by bleomycin or the transient pulmonary overexpression of TGF-β or IL-1β [41–44]. Anti-sense inhibition of HSP27 prevented the stabilization of the TGF-β-induced, pro-EMT transcription factor *Snail*, and thereby allowed for its proteosomal degradation [45]. In a transient overexpression of TGF-β model of pulmonary fibrosis, HSP27 anti-sense treatment inhibited EMT and fibrosis development. Recently, Bellaye et al. [46] showed that a decrease in αB-crystallin protein levels reduced TGF-β signaling by promoting Smad4 mono-ubiquination and nuclear export. αB-crystallin knockout mice were protected against bleomycin-induced pulmonary fibrosis and fibrosis induced by over-expression of TGF-β or IL-1β [46, 47]. Although our in vitro results did not show a Compound A-induced decrease in Smad4 levels after one hour incubation with TGF-β, we may not have observed it because of the time frame examined. These findings on αB-crystallin may point to another biochemical intersection between the inhibition of MAP3K19 activity and TGF-β signaling. Finally, both approved drugs for IPF have been reported to have anti-TGF-β pathway effects. In a retinal epithelial cell line, ARPE-19, very high concentrations of pirfenidone were shown to prevent the nuclear accumulation of phospho-Smad2/3 [48]. The multi-kinase inhibitor, nintedanib, has also been shown to inhibit TGF-β-induced Smad3 phosphorylation [49]. Thus, in both animal models and IPF, inhibition of the TGF-β pathway has been shown to have beneficial therapeutic effects.

In our in vivo studies, inhibition of MAP3K19 by Compound A administered either prophylactically or therapeutically, strongly reduced bleomycin-induced fibrosis and inflammation (Figs 15, 16 and 17). Given that the pulmonary administration of bleomycin has been shown to increase TGF-β gene expression and protein levels [50, 51], these results are mechanistically consistent with other therapeutic efforts to block TGF-β signaling in pulmonary fibrosis. As the current standard of care drugs, pirfenidone and nintedanib have a large side effect profile, the specificity of Compound A coupled with the restricted pattern of expression of MAP3K19 suggest that it may not have these downsides in patients (Figs 1–4, S1 Fig). Taken together, the results reported here suggest that inhibition of MAP3K19 represents a novel and unique approach to target the TGF-β pathway that may have therapeutic benefit for IPF.

## Supporting Information

S1 Fig MAP3K19 has a restricted expression pattern in the body. Human tissue microarrays were stained with either anti-MAP3K19 antibody (RabK19) or a negative control antibody. As shown below, there is strong staining in the lung, less staining in the kidney (some MAP3K19^**+**^ cells are pointed out by black arrows), and no detectable MAP3K19 positive cells in the heart or testis. Interestingly, the testis showed the greatest amount of MAP3K19 mRNA expression as detected by RT-qPCR, however the IHC analysis shows that these mRNA transcripts are not translated into protein. This is in contrast to the lung, which shows high levels of both MAP3K19 mRNA and protein.
(TIF)

S2 Fig MAP3K19 siRNA knocks down MAP3K19 mRNA expression in HeLa cells. RT-qPCR analysis of MAP3K19 expression in HeLa cells either untreated, mock transfected or transfected with 250 ng or 500 ng of either non-sense siRNA or MAP3K19 siRNA. This is a control for the experiment shown in Fig 5A of the manuscript.
(TIF)

S3 Fig Transfection of MAP3K19 expression plasmid into HEK cells increases mRNA expression of MAP3K19. RT-qPCR of MAP3K19 expression in HEK cells that were either untreated, mock transfected with pcDNA3.1 or transfected with a MAP3K19 expression plasmid. RNA was isolated 24 hours post-transfection and subject to RT-qPCR analysis. This is a control experiment for the experiment shown in Fig 5D of the manuscript.
(TIF)

S4 Fig MAP3K19 expression in normal mouse lung, bleomycin treated mice (day 14) and bleomycin and Compound A treated mice (day 14). Similar to human lung expression, MAP3K19 is expressed in bronchial ciliated epithelial cells, alveolar and non-alveolar macrophages, a subpopulation of lymphocytes, and random fibroblasts. There are more MAP3K19 positive cells in the bleomycin treated animals because of the inflammation however the same cell types are positive for expression.
(TIF)
