# Supplementary figures and images for: MAP3K19 Is a Novel Regulator of TGF-β Signaling That Impacts Bleomycin-Induced Lung Injury and Pulmonary Fibrosis
Source: PLoS One. 2016 May 4;11(5):e0154874. doi: 10.1371/journal.pone.0154874 (PMC4856290; doi:10.1371/journal.pone.0154874)

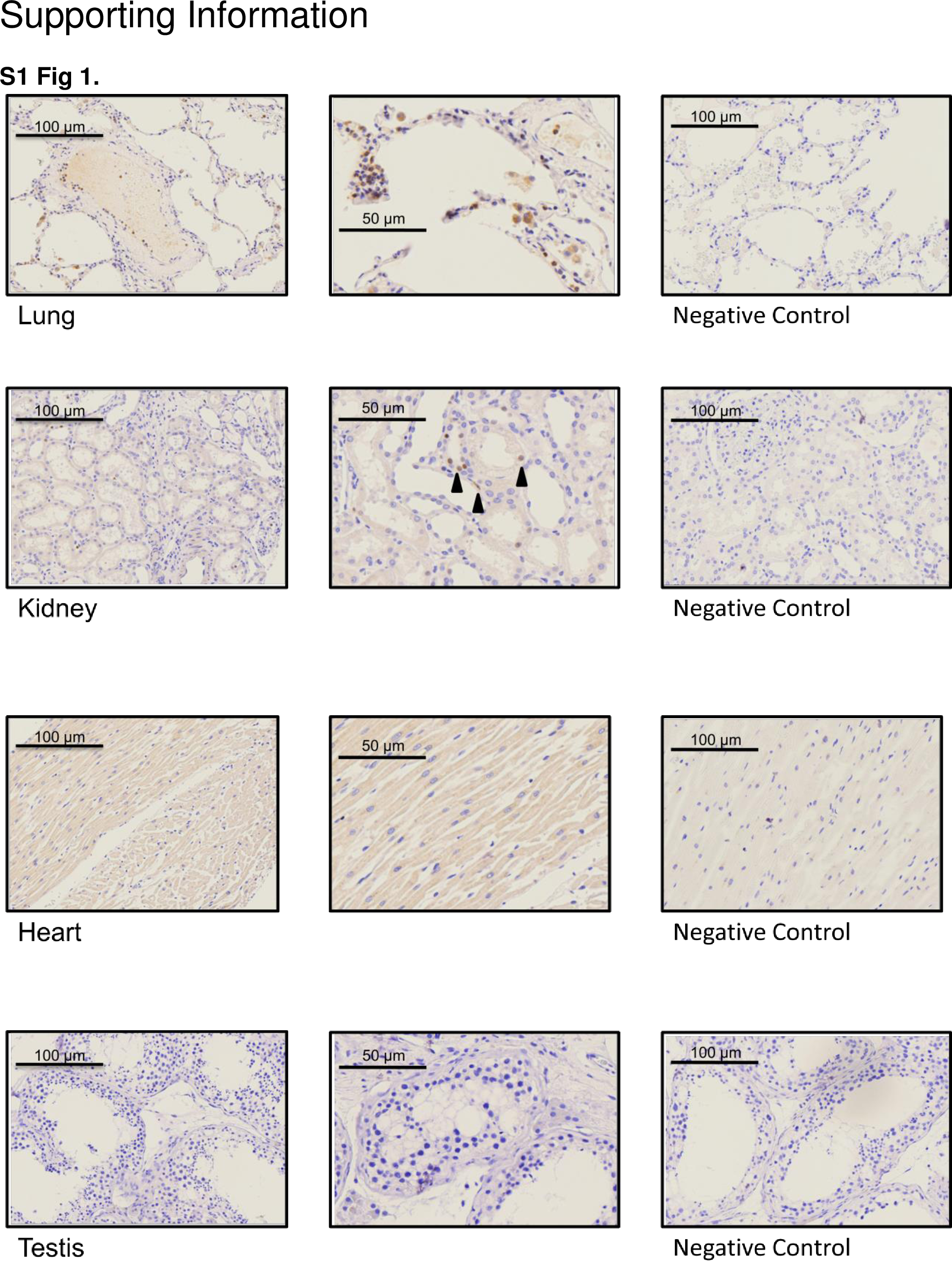

Supplement: S1 Fig — Human tissue microarrays were stained with either anti-MAP3K19 antibody (RabK19) or a negative control antibody. As shown below, there is strong staining in the lung, less staining in the kidney (some MAP3K19+ cells are pointed out by black arrows), and no detectable MAP3K19 positive cells in the heart or testis. Interestingly, the testis showed the greatest amount of MAP3K19 mRNA expression as detected by RT-qPCR, however the IHC analysis shows that these mRNA transcripts are not translated into protein. This is in contrast to the lung, which shows high levels of both MAP3K19 mRNA and protein. (TIF) [file pone.0154874.s001.tif]

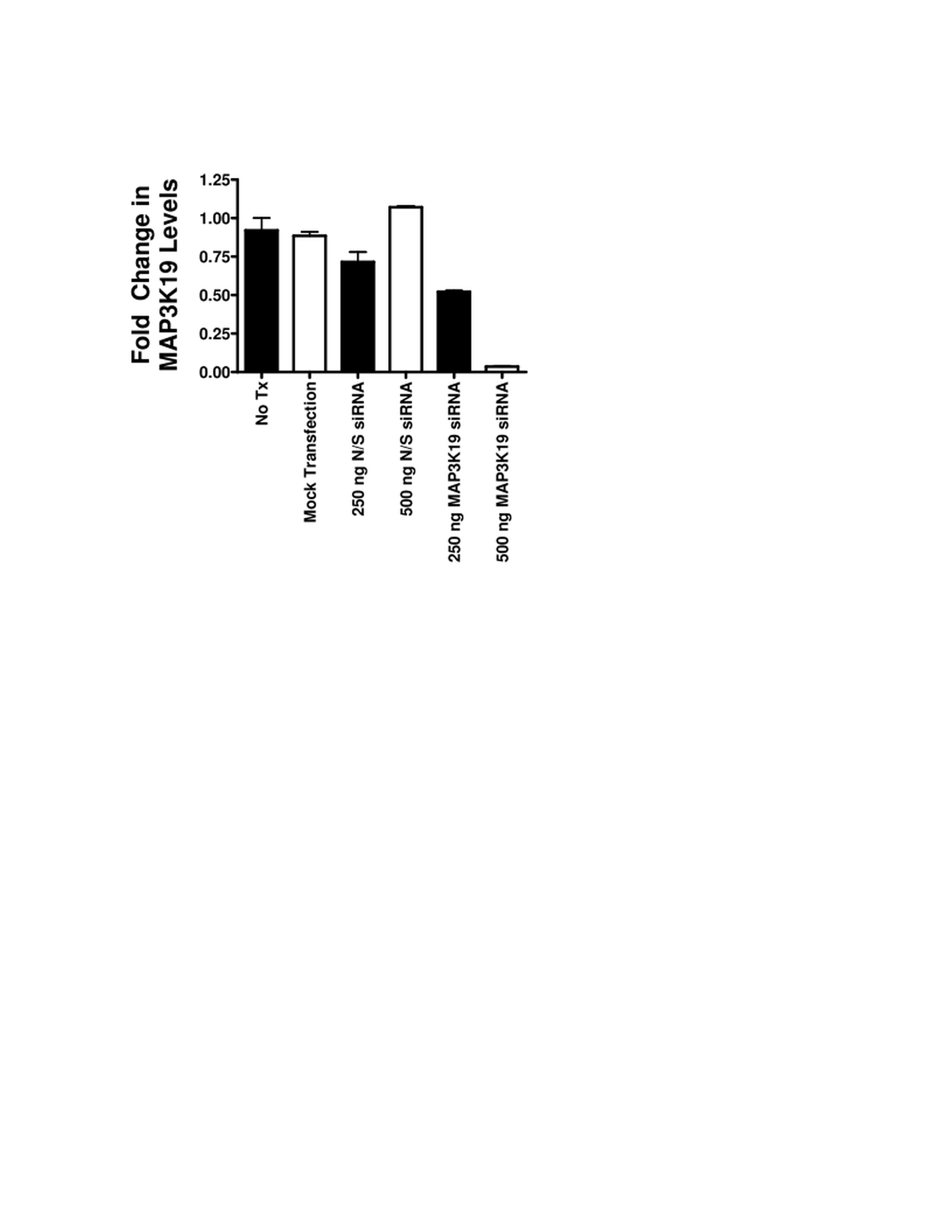

Supplement: S2 Fig — RT-qPCR analysis of MAP3K19 expression in HeLa cells either untreated, mock transfected or transfected with 250 ng or 500 ng of either non-sense siRNA or MAP3K19 siRNA. This is a control for the experiment shown in Fig 5A of the manuscript. (TIF) [file pone.0154874.s002.tif]

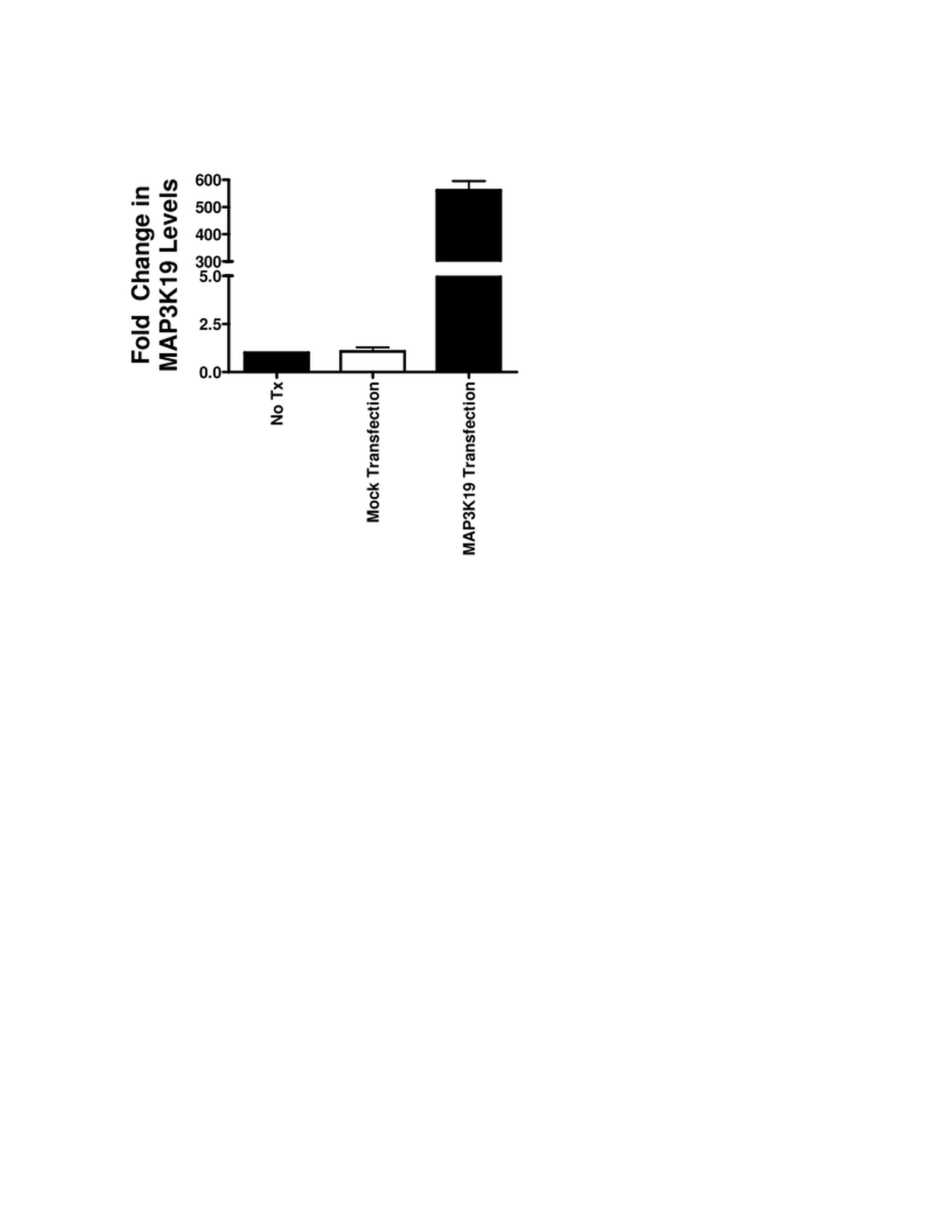

Supplement: S3 Fig — RT-qPCR of MAP3K19 expression in HEK cells that were either untreated, mock transfected with pcDNA3.1 or transfected with a MAP3K19 expression plasmid. RNA was isolated 24 hours post-transfection and subject to RT-qPCR analysis. This is a control experiment for the experiment shown in Fig 5D of the manuscript. (TIF) [file pone.0154874.s003.tif]

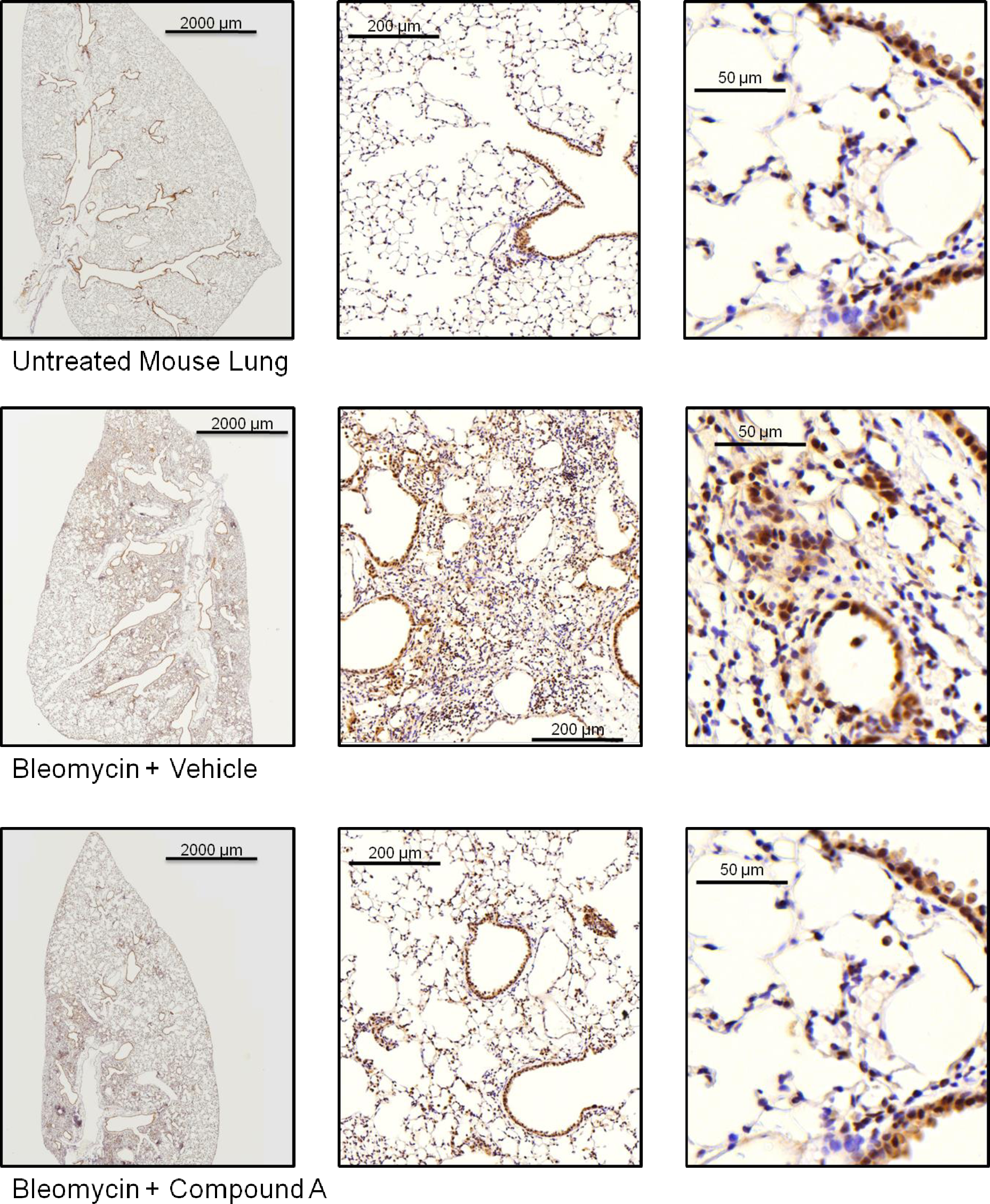

Supplement: S4 Fig — Similar to human lung expression, MAP3K19 is expressed in bronchial ciliated epithelial cells, alveolar and non-alveolar macrophages, a subpopulation of lymphocytes, and random fibroblasts. There are more MAP3K19 positive cells in the bleomycin treated animals because of the inflammation however the same cell types are positive for expression. (TIF) [file pone.0154874.s004.tif]
